# Physiological specialization of *Puccinia triticina* and genome-wide association mapping provide insights into the genetics of wheat leaf rust resistance in Iran

**DOI:** 10.1038/s41598-023-31559-y

**Published:** 2023-03-16

**Authors:** Reza Talebi, Mozghan Mahboubi, Amir Mohammad Naji, Rahim Mehrabi

**Affiliations:** 1 Department of Plant Breeding, Islamic Azad University, Sanandaj Branch, Sanandaj, Iran; 2 Department of Agronomy and Plant Breeding, Faculty of Agriculture, Shahed University, Tehran, Iran; 3 Department of Biotechnology, College of Agriculture, Isfahan University of Technology, PO Box 8415683111, Isfahan, Iran; 4 Present Address: Keygene N.V, Agro Business Park 90, 6708 PW Wageningen, The Netherlands

**Keywords:** Genetics, Plant sciences

## Abstract

Leaf rust caused by *Puccinia triticina* Erikss. (Pt) is the most widely distributed and important wheat disease worldwide. The objective of the present study was to determine the frequency of Iranian Pt races, their virulence to key resistance genes and map quantitative trait loci (QTL) for resistance to different Pt races from 185 globally diverse wheat genotypes using a genome-wide association study (GWAS) approach. The virulence pattern of the 33 *Pt* isolates from various wheat-growing areas of Iran on 55 wheat differentials showed that the FKTPS and FKTTS were relatively frequent pathotypes among the 18 identified races. The weighted average frequency of virulence on the resistance genes *Lrb*, *Lr3bg*, *Lr14b, Lr16*, *Lr24*, *Lr3ka*, *Lr11* and *Lr20* were high (> 90%). However, low virulence on the resistant genes *Lr2a, Lr9*, *Lr19*, *Lr25*, *Lr28* and *Lr29* indicates that these genes are still effective against the pathogen population in Iran at present. GWAS on a panel of 185 wheat genotypes against 10 *Pt* races resulted into 62 significant marker-trait associations (MTAs) belonged to 34 quantitative trait loci (QTL) across 16 chromosomes. Among them, 10 QTLs on chromosomes 1A, 1B, 3B, 3D, 4A, 6D, 7A and 7D were identified as potential novel QTLs, of which four QTLs (*QLr.iau-3B-2, QLr.iau-7A-2, QLr.iau-7A-3* and *QLr.iau-7D-2*) are more interesting, as they are associated with resistance to two or more *Pt* races. The known and novel QTLs associated with different *Pt* races found here, can be used in future wheat breeding programs to recombine different loci for durable resistance against leaf rust races.

## Introduction

Bread wheat (*Triticum aestivum* L.) has been domesticated in Fertile Crescent 10,000 years ago^1^ and is the most important crop in Iran which is widely cultivated in an area of more than six million hectares. It is suggested that both wheat and its pathogens have co-evolved in this area. Leaf rust incited by *Puccinia triticina* Erikss. (Pt) is a macrocyclic foliar disease of wheat and is the most widely distributed worldwide and generally appears in most of the wheat-growing regions of Iran particularly at early and late growing stages^2^. It is believed that the center of origin of *P. triticina* is the Fertile Crescent region, where the natural range of the primary and alternative hosts overlaps^3^. *P. triticina* can cause significant yield losses over large geographical areas and, thus, is considered as a threat of wheat production worldwide^4^. In Iran, it is the second economic important disease on wheat after yellow rust and under conductive epidemic conditions it is estimated that more than 20% of wheat-growing fields are prone to leaf rust infection^5,6^.

Different control strategies are currently available to control Pt, including fungicide application, biological control and employment of resistant genes/cultivars. Timely and accurate application of fungicides is effective in controlling of leaf rust in wheat^7^, but besides the cost of application, fungicides are serious threats to human health and the environment. On the other hand, the reputed use of fungicides may lead to fungicide-resistance in *Pt* isolates circumventing susceptibility to fungicides^7,8^. Therefore, characterization of resistance genes and development of resistant cultivars are the most economical and environmentally safe approaches for controlling leaf rust.

*P. triticina* populations are highly diverse in terms of genetics and virulence pattern, which is driven by the co-evolution of *Pt* strains with various wheat cultivars in wheat-growing areas worldwide, as well as by genetic recombination of *Pt* races and spontaneous mutations^9,10^. High variability in *P. triticina* populations and its high fitness to diverse environmental conditions results in the regular breakdown of the resistance genes and, hence, the implementation of slow rusting along with race-specific resistance genes has been suggested to enhance the durability of resistance in wheat cultivars^4^.

Clearly, successful control of leaf rust disease requires basic knowledge about the diversity and virulence profiles of the pathogen populations gained through race analysis approach is necessary for effective control of leaf rust disease. This is critical for establishing effective breeding programs for durable resistance^11^ . Genetic resistance against leaf rust in wheat is usually related to seedling resistance (referred to all-stage resistance = ASR) and adult-plant resistance (APR)^7,12^. Seedling resistance is qualitative and controlled by single or major genes that mostly are race-specific resistance and associated with hypersensitive response^13,14^. While APR is mostly non-race specific resistance and controlled by several minor effect genes, the accuracy of phenotyping for leaf rust under field conditions can be affected by environmental factors such as temperature, light, inoculum pressure and plant maturity^12,15^.

To date, more than 80 leaf rust resistance genes and QTLs have been identified, of which some were introgressed from durum or bread wheat cultivars and some were originated from wheat wild relatives such as *Aegilops*, *Agropyron*, *Secale*, and *Thiropyrum*^7^. So far, a large number of resistance genes and QTLs have been identified in various wheat genotypes^16,17^. Most of these wheat cultivars and breeding lines, however, are no longer in use because their resistance has been overcome by new virulent Pt races. Therefore, identification of new sources of resistance using different Pt races and implementation of these resistant genotypes into breeding programs are essentially required to control the leaf rust disease^18–20^.

The objectives of the present study were: (i) to determine the distribution of *Pt* races in different wheat growing zones of Iran and to monitor the dynamics and variation of virulence to leaf rust resistance genes, (ii) to characterize the resistance/susceptibility pattern in a worldwide collection of wheat genotypes to 10 different Iranian Pt races at seedling stage, and (iii) to conduct genome-wide association analysis (GWAS) for identifying molecular markers associated with known Lr resistance genes and novel QTLs.

## Results

### *P. triticina* isolates virulence and race identification

Results of phenotypic interaction of 33 single uredinia of *P. triticina* isolates on 55 ‘Thatcher’ near-isogenic lines at the seedling stage presented in Table S1. In total 18 physiological races were identified (Table S1). Among all races, FKTPS (15%) and FKTTS (12%) were the most common pathotypes, which were collected mainly from Khuzestan province (southwest of Iran). Phenotypes LKTTS, PJTSS and PKRQS had an occurrence frequency of 9% each. For each DTRRS and PKRQS phenotypes, two isolates were found, while other phenotypes including BKGSS, BRTRS, CFNPs, CFTTS, CTTPR, MFHPs, MHRRS, MJTTS, MTTTS, PTMQS and FSRRS were represented by single isolates.

Geographic distribution of the leaf rust samples is shown in Table S1. The results showed that similar races like DTRRS, FKTTS, LKTTS, PJTSS and PKRQS were isolated from either a single field or geographically close fields. In contrast, FKTPS phenotypes were found from different long-distance locations. Some fields like (Shavoor) contain several races.

Frequencies of virulence to *Lr* genes or gene combinations were compared. Virulence to *Lr2a* was not found in any studied area while all isolates were virulent on differentials possessing *Lrb*, *Lr3bg* and *Lr14b*. Virulence to *Lr28* was detected only in Kalardasht. Similarly, virulence to *Lr9* was found with a low frequency (21%) and was detected in different geographical locations. Virulence to *Lr1* was Moderate (48%) while the weighted average frequency of virulence to *Lr16*, *Lr24*, *Lr3ka*, *Lr11* and *Lr20* was high frequency (> 90%).

**Figure 1:**
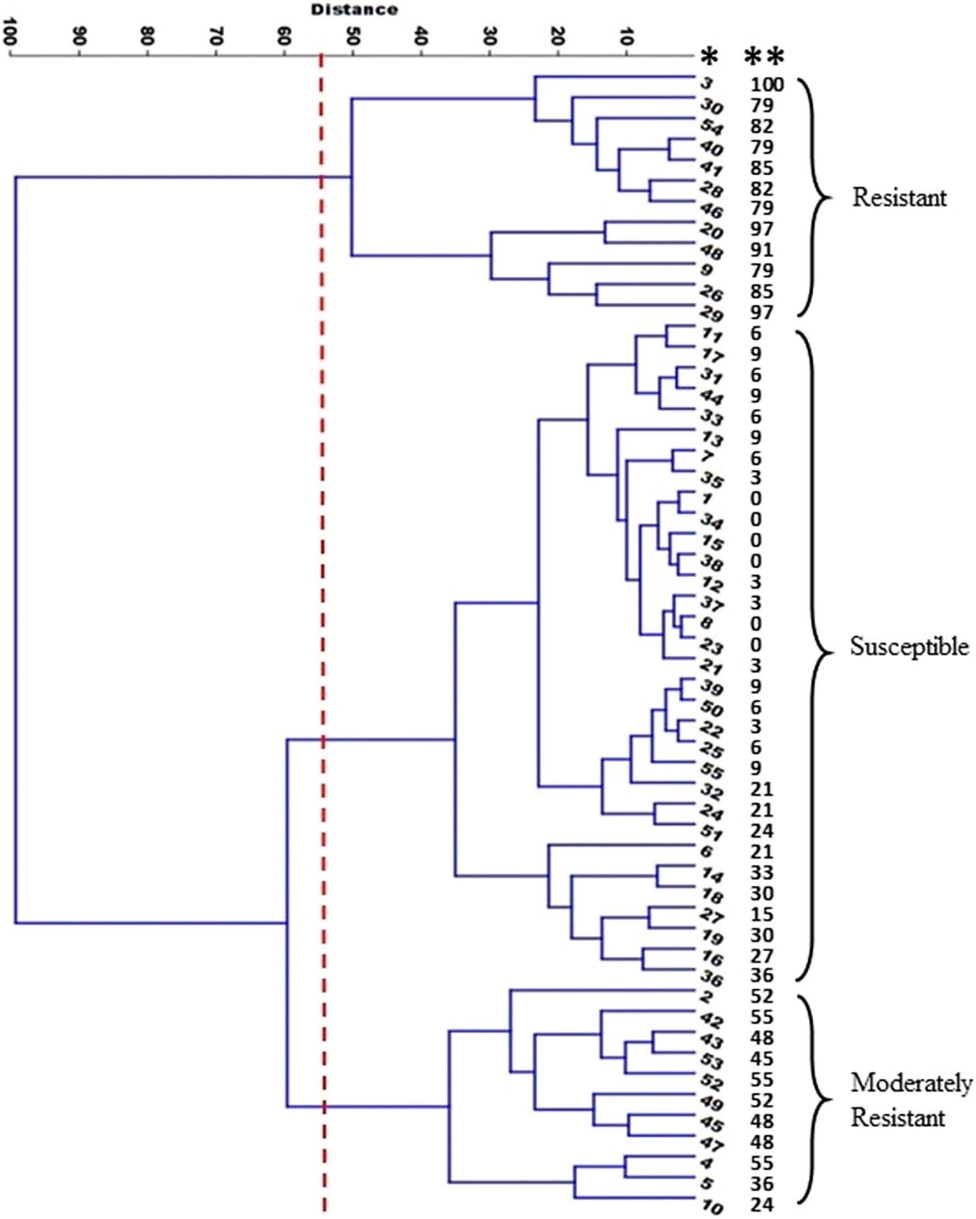
Cluster analysis of 55 wheat differential responses against 33 leaf rust isolates showing that wheat genotypes can be categorized into three major clusters including resistant, susceptible and moderately resistant groups. * genotype number, ** Percentage of avirulent isolates.

Cluster analysis of wheat differentials showed that wheat genotypes can be categorized into three major clusters (Fig. 1). Cluster A consists of 12 wheat genotypes that were considered resistant. Wheat differentials in this cluster had a high frequency of resistance responses with an average of 86% ranging from 79% (*Lr29*, *Lr23* +, *Lr13*/*Lr17*/*Lr27* +/*Lr31*, and *Lr9*) to 100% (*Lr2a*) (Fig. 1). Interestingly, in cluster A, *Lr2a* was resistant to all the tested isolates, which indicates that *Lr2a* is an effective broad resistance gene. Cluster B is consist of 32 wheat genotypes and was identified as a susceptible cluster with a low average resistance response (11%). Six genotypes in this cluster had no specific resistance responses and were susceptible to all the tested isolates. Resistance responses among all cluster members were generally low ranging from 0 to 36%. Lastly, cluster C showed intermediate responses and was considered as a moderately resistant cluster with an average virulence phenotype of 47% ranging from 24 to 55%.

**Figure 2:**
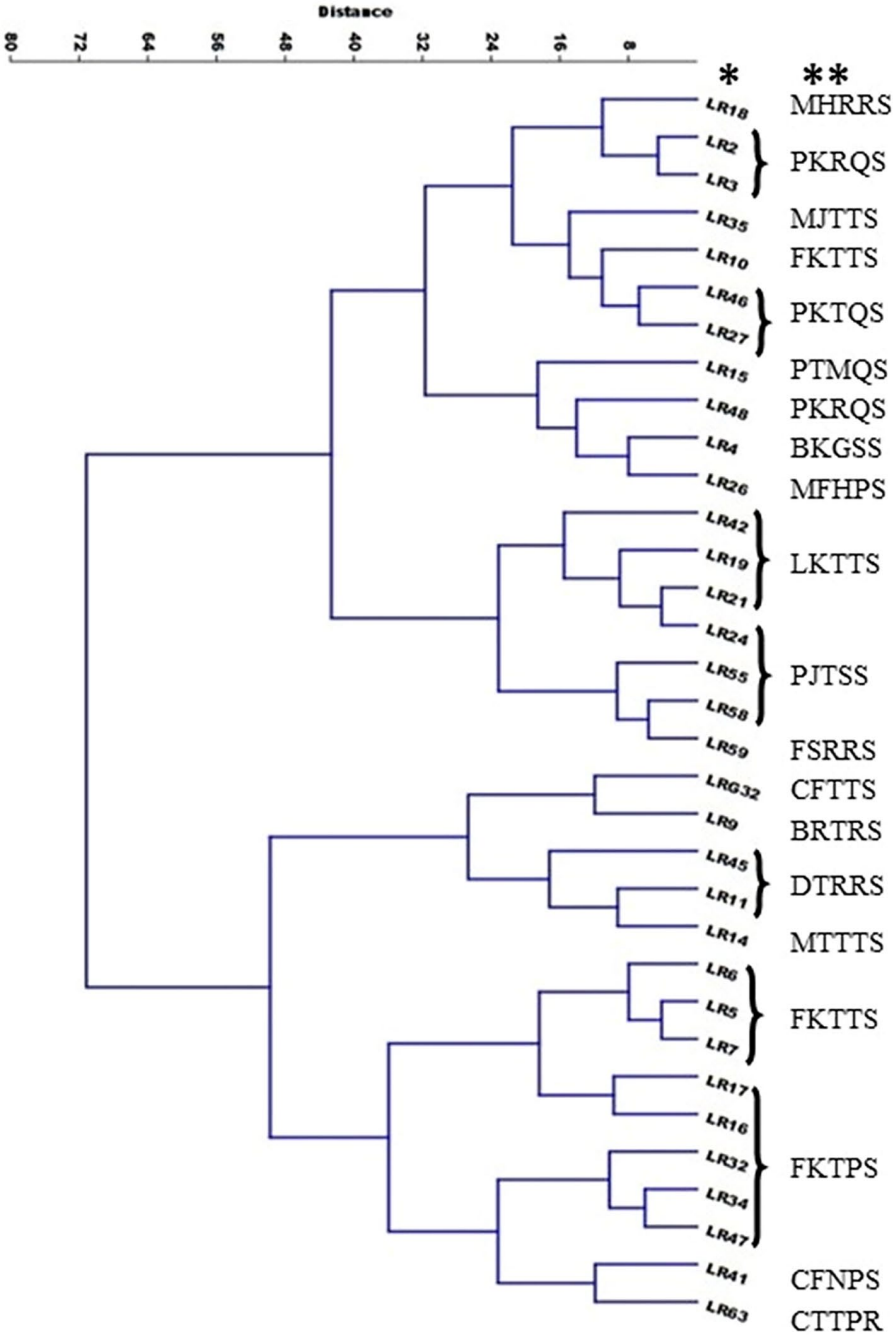
Cluster analysis of 33 isolates according to their virulence spectrum on 55 wheat differentials resulted in several clusters and sub-clusters. Note that the majority of isolates belonging to similar races are grouped in distinct clusters or very close sub-clusters. * Leaf Rust isolates, ** Identified physiologic races.

Cluster analysis of 33 leaf rust isolates according to their virulence spectrum on 55 wheat differentials resulted in two main clusters, and each cluster was divided into two sub-clusters. As expected, similar physiologic races grouped together or into very close sub-clusters (Fig. 2). The only exception were isolates from race FKTTS that were separated in two different locations.

### Wheat germplasm seedling responses to ten Pt races

Infection type (IT) of 185 wheat genotypes against ten Pt races at seedling stage under greenhouse conditions have been presented in Table S2. On the linear scale of 0–9, IT scores ranged from 1 (resistant) to 9 (susceptible), while none of the genotypes showed complete immune responses (IT = 0). Of the ten Pt races, based on IT scores (0–9), less than10% of tested genotypes were found resistant (IT score < 5) to each race, except for FKTPS-1 and FSRRS that 34 (18.3%) and 36 (19.5%) genotypes were resistant, respectively (Table S2). The majority of wheat genotypes used for GWAS analysis showed a high frequency of susceptibility to all races. Based on IT scores, nine genotypes including Oasis (USA), Mehregan (Iran), 40,499 (Australia) and six Iranian advanced breeding lines (ER-M-93-13, ER-N-94-15, ER-S-93-2, ER-S-92-113 and ER-M-92-20) were resistance to all Pt races. Although, an Iranian wheat cv. ‘Parsi’ was resistant to all Pt races except race MTTTS and two landraces (IPK40744 and IPK44673) from USA and India were resistant to all races except races MTTTS and PKRQS (Table S2). Heritability values based on IT scores were high for all Pt races, which means that there was a limited replication variation for phenotypic assessment in relative to genotypic variation. Highly significant positive correlations were observed between Pt races ranging from 0.30 to 0.82, with an average value of 0.56 (Table 1).

**Table 1:** Correlation analysis among the phenotypic data of 185 wheat genotypes evaluated for their reaction to 10 *Pucnina triticina* races.

Variables	MTTTS	PKRQS	PJTSS	MFHPS	MJTTS	FKTPS‐1	CTTPR	FKTPS‐2	FSRRS	BRTRS	Heretability (%)
MTTTS	1										94.12
PKRQS	0.67**	1									93.8
PJTSS	0.56**	0.69**	1								91.17
MFHPS	0.58**	0.66**	0.73**	1							95.14
MJTTS	0.30*	0.41**	0.40*	0.36*	1						93.18
FKTPS-1	0.44*	0.47**	0.38*	0.36*	0.74**	1					92.16
CTTPR	0.50**	0.55**	0.53**	0.59**	0.46*	0.55**	1				92.87
FKTPS-2	0.49**	0.59**	0.68**	0.82**	0.33*	0.35*	0.58**	1			94.16
FSRRS	0.53**	0.64**	0.69**	0.78**	0.42*	0.39*	0.57**	0.82**	1		93.58
BRTRS	0.59**	0.66**	0.72**	0.73**	0.47*	0.45*	0.61**	0.74**	0.79**	1	93.74

* Significant (α = 0.05); ** Significant (α = 0.01)

**Figure 3:**
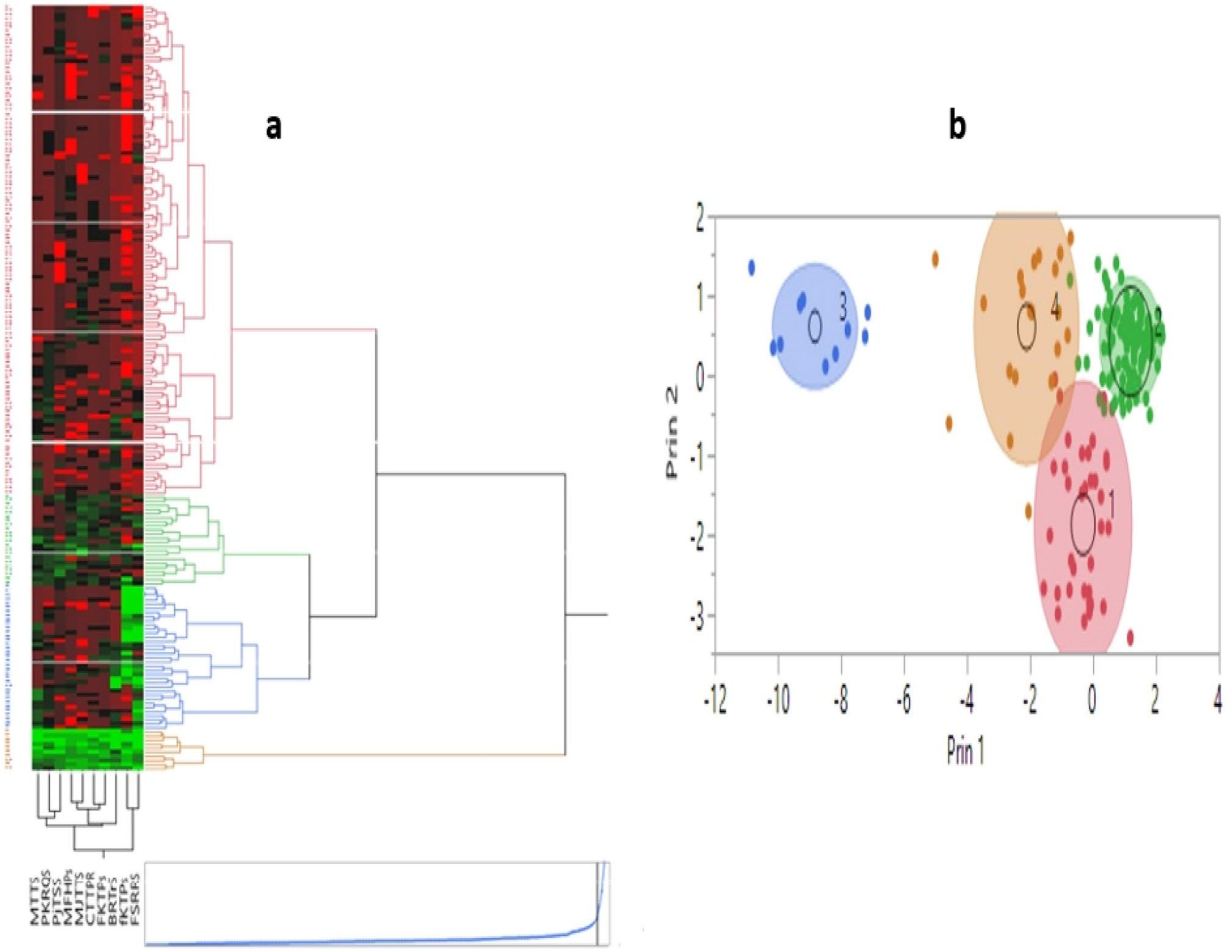
Cluster analysis (**a**) and principal component analysis (PCA) (**b**) of 185 wheat genotypes based on IT scores data against ten *Pucnina. triticina* races.

Cluster analysis and principal component analysis based on IT scores (0–9) grouped 185 wheat genotypes into four clusters (Fig. 3). The first cluster comprised 118 wheat genotypes, which showed high susceptibility to all Pt races (Table 2). Cluster-II contained 22 genotypes, most of which were landraces from different sources and also included four Iranian improved cultivars. None of this cluster genotypes showed high resistance to all races, while a few of them only showed resistant spectra to FKTPS-1, PJTTS or FSRRS races (Table S2). Cluster III comprised 35 genotypes, all of which were Iranian improved cultivars and advanced breeding lines. These genotypes showed resistance spectra to FKTPS-1 and FSRRS races (Table 2). Cluster IV contained 10 genotypes, including six Iranian advanced breeding lines, one Iranian Cultivar ‘Mehregan’, a cultivar originating from the USA ‘Oasis’ and two landraces IPK40499 and IPK44673 from Australia and India, respectively. These genotypes showed a high level of resistance to all Pt races (Table 2; Table S2).

**Table 2:** Means of disease severity (0–9) of wheat genotypes to different *P.triticina* races in four clusters.

Cluster	No. of genotypes	MTTS	PKRQS	PJTSS	MFHPS	FKTPS‐1	FSRRS	BRTRS	MJTTS	CTTPR	FKTPS-2
1	118	7.67	7.53	7.97	7.88	8.08	7.54	7.57	7.82	7.75	7.87
2	22	6.59	5.95	6.64	5.77	6.27	5.68	6.18	5.32	5.95	6.27
3	35	6.66	6.86	7.94	7.91	3.77	2.54	6.17	7.97	7.66	7.43
4	10	3.10	2.90	3.00	2.30	1.90	1.60	2.20	1.90	2.20	2.10

### Wheat panel diversity, population structure and LD analysis

Genotyping of 185 wheat genotypes returned a total of 94,535 raw DArT-seq markers. After marker filtering, in total 21,773 DArTseq markers (including 15,856 SilicoDArT and 5917 SNP) with MAF ≥ 5% and missing data points ≤ 20%, were used for further analysis of population structure, linkage disequilibrium and marker-trait association analysis against 10 *Pt* races. Analysis of genetic diversity using UNJ-clustering and Bayesian model-based structure of 185 wheat genotypes used in this study was previously described by Maboubi et al. (2022), where this wheat panel was grouped into four distinct clusters (Fig. S1). This cluster grouping was relatively consistent with the geographical origin and type (landrace or cultivar) of genotypes. A comparable result similar to population structure and UNJ-clustering was also observed by the heatmap plot of the kinship matrix where four distinct clusters were identified (Fig. S2). The first cluster comprised 50 genotypes, of which 17 were Iranian landraces and as well as landraces originating from Turkey, Romania, Hungary and Tajikistan. All of these genotypes were susceptible to most of the Pt races, except a few Iranian landraces that showed partial resistance to FKTPS-1 race. Cluster-II comprised of 29 genotypes from globally diverse origins and were susceptible to all Pt races. Cluster-III included 86 genotypes, which most of the Iranian cultivars grouped in this cluster. Most of the genotypes in this cluster, with a few exceptions, were resistant to two Pt races (FKTPS-1 and FSRRS). Cluster-IV comprised of 20 genotypes, of which were mostly resistant to multiple Pt races and ten wheat genotypes that showed a high resistance pattern to all Pt races were in this cluster. The results of population genetic diversity were significantly in agreement with phenotypic responses of wheat genotypes against Pt races, with a few exceptions, which indicated the good fit of population structure analysis with phenotypic data that are prerequisite for marker-trait association analysis. In the LD analysis, 28% of the intra-chromosomal pairs showed a significant level (P < 0.001) of the correlation coefficient (r^2^). Mean and critical r^2^ values were 0.09 and 0.16, respectively. Overall, LD between marker pairs decayed quickly in the B genome, followed by the A genome. In the D genome the LD was very pronounced and they did not drop below the critical value over distances of 1.6 kb (Fig. S3).

### Association mapping against Pt races and putative candidate gene identification

The GWAS based on normalized IT scores to ten Pt race/isolate at seedling stages using Farm-CPU model showed reliable results and presented low spurious associations. Association analysis was performed separately for each *Pt* race. A total of 62 significant markers were identified and found distributed across all chromosomes except for 1D, 3A, 4B, 4D and 5D (Table 3 and Fig.S4). The significant markers explained 6–18% of phenotypic variations. The QTLs identified for different *Pt* races but located at an overlapping genomic region on a chromosome were considered a single QTL and assigned the same name using the nomenclature *QLr.iau-* followed by the name and number of QTL in chromosome order and finally, 62 MTAs were assigned to 34 QTL regions on 16 chromosomes (Table 3).

**Table 3:** Summary of the seedling leaf rust resistance quantitative trait loci identified against 10 *Puccinia triticina* races in the panel of 185 wheat genotypes.

QTL	SNP ID	RACE	Chr	Position (bp)	Position (cM)	P value	Annotated gene	Predicted function
QLr.iau‐1A‐1	1,104,046	MJTTS	chr1A	31,875,119	45.11	4.11E − 05	TraesCS1A02G051100	Leucine-rich repeat domain superfamily
QLr.iau‐1A‐2	1,091,963	MFHPS	chr1A	544,082,192	153.82	6.84E − 05	TraesCS1A02G366500	Cytochrome P450
QLr.iau‐1A‐3	3,022,780	MJTTS	chr1A	588,634,744	252.78	5.65E − 05	TraesCS1A02G440300	Leucine-rich repeat-containing N-terminal
QLr.iau‐1B‐1	1,104,236	MJTTS	chr1B	479,758,475	94.37	1.78E − 06	TraesCS1B02G274400	Protein kinase-like domain (Zinc finger, RING/FYVE/PHD-type)
QLr.iau‐1B‐2	1,696,203	MJTTS	chr1B	682,864,419	276.30	7.88E − 05	TraesCS1B02G474600	F-box-like domain superfamily
QLr.iau‐2A‐1	3,938,806	FSRRS	chr2A	6,622,475	9.45	2.61E − 06	TraesCS2A02G016900	P-loop nucleoside triphosphate hydrolase
QLr.iau‐2A‐2	3,027,084	MTTTS	chr2A	24,111,039	65.85	2.25E − 05	TraesCS2A02G057000	Protein kinase-like superfamily (Legume lectin domain)
	4,261,248	PKRQS	chr2A	182,140,338	67.79	1.20E − 06	TraesCS2A02G204700	P-loop containing nucleoside triphosphate hydrolase (Plant myosin class VIII)
	3,021,874	FKTPS‐2	chr2A	389,439,783	68.56	3.22E − 05	TraesCS2A02G255300	Leucine-rich repeat domain superfamily
QLr.iau‐2A‐3	981,785	MFHPS	chr2A	742,781,080	110.47	4.32E − 05	TraesCS2A02G520500	Cytochrome P450 superfamily
	3,024,004	CTTPR	chr2A	769,344,169	122.19	1.39E − 06	TraesCS2A02G573500	Haem peroxidase superfamily
QLr.iau‐2B‐1	995,662	MTTTS	chr2B	18,319,883	13.54	7.24E − 05	TraesCS2B02G038400	Leucine-rich repeat-containing N-terminal, plant-type
	3,532,895	MTTTS	chr2B	28,176,051	24.84	3.10E − 05	TraesCS2B02G058900	Leucine-rich repeat domain (Virus X resistance protein-like)
	3,020,982	PJTSS	chr2B	48,007,814	32.82	4.31E − 05	TraesCS2B02G085400	Leucine-rich repeat domain superfamily
QLr.iau‐2B‐2	1,027,810	MFHPS	chr2B	551,637,948	76.50	9.73E − 05	TraesCS2B02G388800	(Legume lectin domain)
	1,862,545	PKRQS	chr2B	623,869,418	77.50	4.88E − 05	TraesCS2B02G430900	Plant Peptidase S10
	3,946,214	FKTPS‐2,	chr2B	642,834,436	78.31	4.10E − 05	TraesCS2B02G450200	Cytochrome P450
QLr.iau‐2B‐3	1,152,655	CTTPR, MFHPS, MJTTS	chr2B	712,050,374	128.14	2.38E − 06	TraesCS2B02G517300	Leucine-rich repeat domain superfamily
QLr.iau‐2D‐1	3,940,894	MTTTS	chr2D	5,283,967	6.06	8.29E − 05	TraesCS2D02G012800	Cytochrome P450
QLr.iau‐3B‐1	1,088,335	CTTPR	chr3B	518,350,866	60.83	7.43E − 09	TraesCS3B02G320700	Leucine-rich repeat domain superfamily
	1,231,107	CTTPR	chr3B	653,810,881	73.15	4.09E − 05	TraesCS3B02G418800	F-box-like domain superfamily
	5,369,257	FKTPS‐2	chr3B	671,161,431	78.81	1.79E − 05	TraesCS3B02G433100	Leucine-rich repeat domain superfamily
	1,216,374	FKTPS‐2	chr3B	671,161,607	78.81	7.11E − 05	–	–
QLr.iau‐3B‐2	1,229,647	PJTSS	chr3B	797,786,785	131.01	4.27E-05	TraesCS3B02G565900	Leucine-rich repeat domain superfamily
	1,076,425	BRTRS	chr3B	812,658,362	138.38	5.67E − 05	TraesCS3B02G586500	Cytochrome P450
	1,057,473	MFHPS	chr3B	812,993,842	145.09	5.83E − 05	–	–
	1,095,941	MFHPS	chr3B	813,391,786	145.09	4.35E − 05	TraesCS3B02G587400	Leucine-rich repeat domain superfamily
	4,989,676	MFHPS	chr3B	824,481,249	156.69	3.29E − 06	TraesCS3B02G606700	Leucine-rich repeat domain superfamily
	1,111,693	CTTPR	chr3B	827,961,228	156.74	9.02E − 05	TraesCS3B02G609300	F-box-like domain superfamily
QLr.iau‐3D‐1	1,003,778	PKRQS	chr3D	544,419,623	89.14	1.17E − 05	TraesCS3D02G430100	Leucine-rich repeat domain superfamily
QLr.iau‐4A‐1	3,936,450	BRTRS	chr4A	153,654,485	26.46	1.27E − 06	TraesCS4A02G123700	P-loop nucleoside triphosphate hydrolase
	5,967,805	MFHPS	chr4A	202,953,887	27.42	6.14E − 05	–	–
QLr.iau‐4A‐2	995,761	MFHPS, PJTSS,PKRQS	chr4A	607,270,056	54.26	7.25E − 06	TraesCS4A02G318300	Leucine-rich repeat domain superfamily
QLr.iau‐4A‐3	1,351,280	FKTPS‐2	chr4A	629,433,955	88.61	3.96E − 05	TraesCS4A02G355800	Cytochrome P450
QLr.iau‐4A‐4	1,233,446	CTTPR	chr4A	708,659,775	116.03	1.40E − 06	TraesCS4A02G438700	Leucine-rich repeat domain superfamily
QLr.iau‐5A‐1	1,703,104	PJTSS	chr5A	436,890,364	42.45	6.60E − 06	TraesCS5A02G222200	Leucine-rich repeat domain superfamily
	2,277,102	MJTTS	chr5A	503,499,615	54.79	1.14E − 05	TraesCS5A02G294800	Protein kinase-like domain superfamily
QLr.iau‐5B‐1	1,067,819	PJTSS	chr5B	6,391,001	0.00	8.27E − 05	TraesCS5B02G004500	Cytochrome P450
QLr.iau‐5B‐2	4,261,927	CTTPR	chr5B	330,119,186	29.60	1.97E − 06	TraesCS5B02G181000	F-box-like domain superfamily
QLr.iau‐5B‐3	4,911,101	MFHPS	chr5B	528,472,570	54.18	1.93E − 05	TraesCS5B02G341300	F-box-like domain superfamily
QLr.iau‐5B‐4	1,067,151	PJTSS	chr5B	704,868,810	139.40	4.04E − 07	TraesCS5B02G554300	Leucine-rich repeat domain superfamily
	2,258,090	PKRQS	chr5B	706,684,737	146.28	4.80E − 06	TraesCS5B02G560200	Leucine-rich repeat domain superfamily
QLr.iau‐6A‐1	3,064,900	MJTTS	chr6A	39,002,398	33.61	4.83E − 05	TraesCS6A02G071900	Serine-threonine/tyrosine-protein kinase
	1,045,339	BRTRS	chr6A	371,813,592	48.63	6.22E − 05	–	–
QLr.iau‐6B‐1	2,276,989	MFHPS	chr6B	366,585,056	30.36	6.20E − 05	–	–
	985,117	FKTPS	chr6B	485,290,761	30.95	2.12E − 05	TraesCS6B02G269500	Cytochrome P450 superfamily
	1,003,530	CTTPR	chr6B	581,549,680	33.12	1.56E − 06	–	–
	996,529	PJTSS	chr6B	669,396,201	49.88	5.67E − 05	TraesCS6B02G394600	Leucine-rich repeat domain superfamily
QLr.iau‐6D‐1	992,973	PJTSS	chr6D	5,175,026	7.39	3.28E − 05	TraesCS6D02G012900	Leucine-rich repeat domain superfamily
QLr.iau‐7A‐1	3,021,075	CTTPR	chr7A	39,176,390	28.66	1.81E − 08	TraesCS7A02G074600	hydrolase (Helicase superfamily)
	1,102,645	CTTPR	chr7A	107,080,713	39.64	1.21E − 05	TraesCS7A02G155000	Cytochrome P450 superfamily
QLr.iau‐7A‐2	5,373,057	FKTPS‐2, PJTSS	chr7A	301,167,038	88.73	4.79E − 05	–	–
	1,094,354	MTTTS	chr7A	651,881,285	97.58	1.60E − 05	TraesCS7A02G455800	Protein kinase-like domain superfamily (Serine/threonine-protein kinase)
QLr.iau‐7A‐3	1,103,172	MJTTS	chr7A	705,917,325	149.56	4.15E − 05	TraesCS7A02G522300	Cytochrome P450 superfamily
	1,109,797	BRTRS	chr7A	724,430,683	153.97	7.58E − 05	TraesCS7A02G550000	Leucine-rich repeat domain superfamily
QLr.iau‐7B‐1	2,275,239	CTTPR	chr7B	210,779,344	42.30	7.18E − 08	TraesCS7B02G157000	P-loop nucleoside triphosphate hydrolase (Phosphoribosyltransferase-like)
	1,019,331	BRTRS, FSRRS	chr7B	220,270,676	45.11	4.85E − 05	TraesCS7B02G162500	Protein kinase-like domain superfamily
	1,066,279	FKTPS	chr7B	263,698,243	-	3.85E − 05	TraesCS7B02G179000	Papain-like cysteine peptidase superfamily
QLr.iau‐7B‐2	1,017,404	FKTPS	chr7B	688,713,574	99.89	1.67E − 05	TraesCS7B02G419600	Leucine-rich repeat domain superfamily
QLr.iau‐7D‐1	4,910,573	FKTPS‐2	chr7D	41,889,844	41.31	1.32E − 05	TraesCS7D02G072300	P-loop nucleoside triphosphate hydrolase (Kinesin-like protein)
QLr.iau‐7D‐2	1,079,705	FKTPS‐2	chr7D	420,047,431	100.64	4.01E − 06	TraesCS7D02G331500	Leucine-rich repeat domain superfamily
	3,959,264	MTTTS	chr7D	458,680,743	104.63	9.28E − 05	TraesCS7D02G354700	Leucine-rich repeat-containing N-terminal, plant-type

Most of significant regions (QTLs) were associated with resistance to multiple races, although 18 QTLs showed race-specific resistance on chromosome 1A (*QLr.iau-1A-1, QLr.iau-1A-2* and *QLr.iau-1A-3*), IB (*QLr.iau-1B-1* and *QLr.iau-1B-2*), 2A (*QLr.iau-2A-1*), 2B (*QLr.iau-2B-3*), 2D (QLr.iau-2D-1), 3D (*QLr.iau-3D-1*), 4A (*QLr.iau-4A-2*, *QLr.iau-4A-3* and *QLr.iau-4A-4*), 5B (*QLr.iau-5B-1*, *QLr.iau-5B-2* and *QLr.iau-5B-3*), 6D (*QLr.iau-6D-1*), 7B (*QLr.iau-7B-2*) and 7D (*QLr.iau-7D-1*) (Table 3). The large effect loci on chromosomes 2A, 2B, 3B, 4A, 5B and 7A were associated with responses to multiple *Pt* races. Resistance-associated QTLs localized on 8 and 7 different chromosomes were identified for CTTPR and MFHPS races, respectively, while for FSRRS/FKTPS and BRTRS races, resistance-associated QTLs were identified only on two and three chromosomes, respectively (Table 3). In this study, two isolates belonging to FKTPS race were used. For FKTPS-2 (originated from Ahvaz, southwest of Iran) multiple QTLs localized on six different chromosomes were identified. Interestingly, all QTLs against FKTPS-1 (originated from Neishaboor, north-east of Iran) were different from those QTLs identified for FKTPS-2.

The chromosomal position of MTAs associated with resistance to Pt races were mapped to the Chinese Spring cv. wheat physical genome. For each MTA, 2.5 Mb region toward the left and right side was used to identify the putative candidate genes. Totally, in 56 MTAs we identified several putative candidate genes previously known to play a role in defense mechanisms such as genes encoding leucine-rich repeat (LRR), protein kinase, zinc finger and P-loop-NTPase proteins (Table 3).

## Discussion

### Pathotypes and physiologic specialization of Iranian wheat leaf rust

Wheat is the most important cereal food crop worldwide and it has been domesticated and cultivated in Iran from ancient times^2^. Leaf rust caused by *P. triticina* Eriks (Pt), is the most important and common foliar disease of wheat in Iran and most wheat growing area worldwide^3,21^. Given the fact that both wheat and *P. triticina* have coevolved in Near-East as well as in Iran, so this fungi has probably been present in this area for thousands of years^3^.

In this study, 33 *P. triticina* isolates from different wheat-growing areas of Iran were collected and tested for race determination based on their reaction on 55 differential wheat genotypes possessing different *Lr* resistance genes. Our results showed that most of the resistance genes were ineffective against *P. triticina* population. However, low virulence phenotype on *Lr2a, Lr9*, *Lr19*, *Lr25*, *Lr28*, and *Lr29* indicates that these genes are still effective against the wheat leaf rust population in Iran at present, which is basically consistent with previous studies^3,22,23^. For example, leaf rust surveys conducted in Iran from 2002 to 2004 indicated no virulence for *Lr9, Lr18, Lr19, Lr25, Lr28, Lr29, Lr34, Lr35, Lr36*, or *Lr37* in the field^22^. In addition, no virulence to *Lr2a, Lr3ka, Lr9, Lr14a, Lr19, Lr23, Lr25, Lr26, Lr28, Lr29, Lr30, Lr32,* or *Lr36* was detected in 2008. Furthermore, race analysis in 2009 and 2010 showed that virulence to *Lr9*, *Lr28*, *Lr25*, *Lr19*, *Lr29*, and *Lr2a* were at low frequencies^23^.

It is believed that forces of mutation, migration, sexual and asexual recombination and selection pressure play significant roles in pathogenic diversity and appearance of new races of rust diseases^24^. Like other rust diseases, urediniospores of leaf rust could migrate thousands of kilometers causing exotic races and clonal reproduction^3,25^.

Recent studies report the similarity between some Iranian and Russian *P. triticinia* isolates that might be attributed to northerly winds that blow from Russia to the north of Iran^2^. On the contrary, our results showed large difference between the phenotypes of Iranian and Russian isolates. None of the virulence phenotypes across Russia had virulence on the leaf rust resistance genes *Lr24* or *Lr28* and phenotypes with virulence on *Lr16* and *Lr18* were at frequencies < 10% of total isolates and were not present in all regions. Interestingly, unlike Iranian isolates that were avirulent to *Lr2a¸*virulence phenotypes on this gene was found at high frequency (66%)^26^. In Pakistan virulence to *Lr9*, *Lr19* or *Lr28* was not identified^27^ that is partly similar to what was observed in this study. However, recently virulence on *Lr2a* was identified^28^, which is different from Iranian isolates. In other neighboring countries like Armenia, Azerbaijan, Tajikistan, Kazakhstan, Uzbekistan, and Kyrgyzstan that are located in North of Iran (Central Asia), no virulence on *Lr9*, *Lr23*, *Lr24* or *Lr26* was found^29^. While no virulence’s were detected against *Lr12*, *Lr15*, *Lr17, Lr22a* and *Lr24* in Iraq^30^*.* In Syria, no virulence for *Lr1*, *Lr2a*, *Lr9*, *Lr15*, *Lr19*, *Lr21*, *Lr24*, *Lr25*, *Lr26*, *Lr28*, or *Lr29* was observed in greenhouse tests showing that Syrian isolates were less aggressive than those of leaf rust isolates in this region^31^.

This section of our findings provided detailed information on the variation in virulence patterns of Iranian *P. triticina* isolates, a country located in the Fertile Crescent where wheat domestication began and coincided with the speciation and further evolution of its pathogens^32^. We demonstrate that *P. triticina* isolates have a broad virulence spectrum against most of the known *Lr* genes indicating that extensive host adaptation has occurred in *P. triticina* populations during the synchronic domestication process of both host and pathogen in this region. Our data shed light into the potential employment of the effective *Lr* genes like *Lr2a, Lr28* and *Lr19* that are of interest to wheat breeding programs to improve the resistance of Iranian wheat cultivars against leaf rust.

### Novel resistance sources and alignment with previously reported QTLs and *Lr* genes

Characterization of novel resistance sources is the prerequisite and most important strategy for controlling rust diseases in wheat and pyramiding these genes for durable resistance^33,34^. The rapid evolution of pathogens due to fungicide application, environmental conditions and also narrow genetic base of resistance genes in improved wheat genotypes can easily lead to the breakdown of the resistance genes^17,35^. In this study, 185 wheat genotypes comprising Iranian cultivars and landraces from diverse world geographical origins were evaluated for resistance against 10 Pt races at seedling stages. Based on IT scores, only three cultivars (Oasis, Mehregan and Parsi) and six Iranian advanced breeding lines showed resistance to all Pt races. Interestingly, all of these genotypes originated from Iran, except for Oasis originated from the USA. The resistance frequency of wheat genotypes for most of the *P. triticina* races was very low (~ 15%), except for two races (FKTPS-1 and FSRRS). Therefore, the resistance pattern of the studied wheat germplasm did not correspond to the virulence profiles of the *P. triticina* races as we identified on the set of 55 wheat differential genotypes. It can be concluded that these wheat genotypes may carry multiple previously known *Lr* genes or in combination with new genes^17,36^.

Significant positive correlations were observed for infection types of ten *P. triticina* races (Table 1). This can conclude that by pathogenicity test results of these ten races on a set of 55 differential genotypes (Table S1), in which all Pt races were virulent on *Lr3*, *Lr11*, *Lr12*, *Lr13*, *Lr20*, *Lr21*, *Lr33*, *Lr34*, *Lr35* and *Lr37*. On the other hand, it is likely that the wheat panel used for the GWAS had multiple genomic loci conferring resistance to multiple races, which was further confirmed by the results of association mapping analysis (Table 3). Similar results for significant phenotypic correlation between multiple races of *P. triticina* in different GWAS panel have been reported^14,37,38^. Therefore, to elucidate the genetics of resistance to *P. triticina* in the wheat panel we implemented a high-throughput genome association analysis using DArTseq markers against 10 Pt races.

Overall GWAS analysis using different races identified 62 MTAs that were assigned to 34 QTL regions on 16 chromosomes (Table 3). these genomic regions were compared with the previously known leaf rust resistance (*Lr*) genes and QTLs projected on consensus maps^39,40^ (Fig. S5). Three QTLs on chromosome 1A were race-specific for resistance to MJTTS and MFHPS races. Two QTLs (*QLr.iau-1A-1* and *QLr.iau-1A-2*) co-located with previously known adult plant resistance (APR) QTLs^41–43^. The QTL *Qlr.iau-1A-3* (252.78 cM) was detected for resistance to MJTTS race and did not align with any previously reported QTL or *Lr* genes, therefore it considered as a potential novel QTL. Two QTLs on chromosome 1B were race-specific for resistance to MJTTS including *QLr.iau-1B-1* was co-localized with different previously known APR resistance QTLs^44–48^ as well as with four known resistance genes *Lr33*, *Lr44*, *Lr71* and *Lr75*^49^. The QTL *QLr.iau-1B-2* (276.30 cM) mapped on 1BL chromosome, but its chromosomal location is far (50 cM) from recently reported APR resistance QTL as well as a QTL found against THBL race form the USA on this chromosome arm^12,50^. Therefore, this region can be considered as a novel locus for race-specific resistance to MJTTS. Both of the QTLs on chromosome 1B were associated with the resistance to MJTTS, but localized on different arms.

Three QTLs were detected on chromosome 2A, of which *QLr.iau-2A-1* was race-specific for resistance to FSRRS and the other two QTLs were detected against multiple races. All these QTLs were co-localized with previously known APR resistance QTLs^12,44,51,52^. Three QTLs were detected on chromosome 2B, of which two QTLs (*QLr.iau-2B-1* and *QLr.iau-2B-2*) were associated with multiple Pt races and co-localized with previously known resistance genes (*Lr18* and *Lr37*) and QTLs^53^. *QLr.iau-2B-3* was race-specific for resistance to MJTTS and co-localized with previously known QTLs at the adult plant stage^47,54^.

Two genomic loci were detected on chromosome 3B, of which *QLr.iau-3B-1* was associated with resistance to two races, CTTPR and FKTPS. This QTL co-localized with previously known QTLs associated with APR resistance in the field^46,55,56^. Interestingly, another QTL (*QLr.iau-3B-2*) was associated with resistance to multiple races (PJTSS, BRTRS, MFHPS and CTTPR) and did not align with any previously reported QTL or *Lr* genes, therefore we assume this QTL might be as a potential novel QTL. The *QLr.iau-3D-1* QTL was associated with the race-specific resistance to PKRQS race and did not align with any previously QTLs on this chromosome. Given the fact that no resistance *Lr* gene except a few QTL for leaf rust resistance identified on chromosome 3D, further studies are needed to elucidate these loci for resistance to more races and also for finding the exact position with more closely significant markers in this region.

Four genomic loci were detected on chromosome 4A, of which two QTLs (*QLr.aiu-4A-1* and *QLr.aiu-4A-2*) were associated with multiple races and co-localized with previously known QTLs and *Lr30*^57,58^. In addition, two QTLs, *QLr.aiu-4A-3* and *QLr.aiu-4A-4* were associated with race-specific resistance to FKTPS and CTTPR, respectively. These genomic loci were not aligned with any previously identified QTLs or *Lr* genes, therefore, we concluded that these are potential novel QTLs. Four race-specific genomic loci on chromosome 5B were identified to be associated with different races and three QTLs on chromosomes 5A, 6A and 6B were associated with resistance to multiple races. All these QTLs co-localized with previously known QTLs for adult plant resistance^42,47,52,55,58–60^.

A QTL *QLr.iau-6D-1* on chromosome 6D was associated with race-specific resistance to PJTSS and did not align with previous reported QTLs on this chromosome. So far no *Lr* gene has been identified, and only a few QTLs for leaf rust resistance have been identified on this chromosome^45^, which did not align with *QLr.iau-6D-1* indicating that *QLr.iau-6D-1*is a potential novel QTL, which needs to be further investigated.

Three QTLs were identified on chromosome 7A, of which QTL *QLr.aiu-7A-1* was associated with resistance to CTTPR and FKTPS races and co-localized with previously reported adult plant resistance QTLs reported^12^ and *Lr47*, which is a seedling leaf rust resistance gene that introgressed from *Triticum speltoides* into the bread wheat genome^61^. Another two QTLs, *QLr.iau-7A-2 and QLr.iau-7A-2* were also associated with resistance to multiple races and did not align with previously known QTLs or *Lr* genes, which can be considered as potential novel QTLs. Two QTLs on chromosome 7B were associated with resistance to multiple races and co-localized with previously known APR resistance QTLs^42,62^. Two QTLs identified on chromosome 7D, of which *QLr.iau-7D-1* co-localized with previously reported leaf rust QTLs^63,64^. QTL *QLr.iau-7D-2* associated with resistance to FKTPS and MTTTS races mapped at a distance of ≥ 20 cM from *L34*. According to the pathogenicity test of ten *P. triticina* races used in this study on 55 differential genotypes, all of them were virulent to *Lr34*. Therefore, *QLr.iau-7D-2* is unlikely to be *Lr34*, which can be considered as a novel QTL.

## Conclusions

High numbers of *P. triticina* races detected in this study from different wheat growing areas in Iran showed a relatively high diversity of *Pt* isolates/races that could be due to migrations of this pathogen from neighboring countries like Russia, Turkey and Iraq to Iran. Different virulence patterns of these isolates against wheat differentials indicated that some *Lr* genes like *Lr2a*, *Lr9*, *Lr25*, *Lr28* and *Lr29* are still effective against Iranian *Pt* races and can be used in breeding programs. Results of GWAS analysis on 185 worldwide wheat genotypes using 10 *Pt* races, identified 34 QTLs, of which 18 were race-specific and 14 QTLs were associated with resistance to two or more *P. triticina* races. Consequently, 10 loci on chromosomes 1A, 1B, 3B, 3D, 4A, 6D, 7A and 7D were identified as potential novel QTLs. Four of those (*QLr.iau-3B-2, QLr.iau-7A-2, QLr.iau-7A-3* and *QLr.iau-7D-2*) are more interesting, as they are associated with resistance to two or more *Pt* races. Most of the identified QTLs in this study were co-localized with previously known APR resistance QTLs. Our finding can be used for combining seedling resistance with APR QTLs/genes, which is an effective and promising strategy for durable leaf rust resistance in wheat.

## Materials and methods

### *P. triticina* isolation and propagation

During the spring and summer of 2016, naturally infected wheat fields from 10 provinces and 18 distant wheat-growing locations were surveyed. In general from each location 2–4 leaf rust samples were collected, air-dried, and temporarily stored at 4 °C in a refrigerator until later use (Table S1). From each sample a single *P. triticina* uredinia was isolated, purified and used for further investigation. To do so, the dried leaves were placed on wet filter papers in Petri dishes and kept at 20 °C overnight. Uredinia were then inoculated onto 10-day-old seedlings of Iranian susceptible cv. Boolani. After inoculation, wheat plants were then transferred to a dark room overnight at %100 relative humidity (25 °C) and then were returned and maintained in a greenhouse at 20–25 °C with supplemental fluorescent lighting to provide a photoperiod of 16 h with a light density of 16,000 lx. Single pustules were derived from each sample after 14 days post inoculation and were increased on susceptible seedling plants again using the same procedure. Isolates were collected by vacuum collectors or by tapping wheat leaves having uredinia. Uredinia were dried in a desiccator containing silica gel for two days and stored at − 80 °C for later use.

### Race and virulence identification

The first experiment for *P. triticina race* and virulence identification was carried out at the Cereal Research Department, Seed and Plant Improvement Institute (SPII), Karaj, Alborz, Iran, in 2018. This experiment included 33 *P. triticina* isolates, which were tested on 55 near-isogenic Thatcher wheat lines (each comprising a single resistance gene). These included set 1: *Lr1*, *Lr2a*, *Lr2c* and *Lr3*, set 2: *Lr9*, *Lr16*, *Lr24* and *Lr26*, set 3: *Lr3ka*, *Lr11*, *Lr17* and *Lr30*^65^, set 4: *Lrb*, *Lr10*, *Lr14a* and *Lr18*^66^ and set 5: *Lr3bg*, *Lr14b*, *Lr20* and *Lr28*^13^. In addition, a set of other resistant lines each possessing multiple *Lr* genes in the different genetic background was used (Table S1). To conduct virulence assay, three pots (as a three replications) of each genotype contained 6–10 seeds in each pots were planted and 10-days old seedling plants were inoculated with uredinia of each isolate suspended in mineral oil (0.3 ml L^−1^) at the concentration of 6 × 10^5^ spores/ml^67^.

The infection types (IT) on the primary leaves were recorded at 14 days post-inoculation, when uredinia on susceptible cultivar were fully developed using 0-to-4 scaling system as described previously^24,68^. Infection types 0 to 2 + were considered to show avirulence for a particular *Lr* gene and infection types 3 to 4 virulence. Based on the low or high infection types of each isolate on the 55 wheat near-isogenic Thatcher lines, a five-letter code for each race was designated using the North American letter code nomenclature system^69^. Cluster analysis of IT data for both wheat differentials and isolates was done based on the dissimilarity matrix calculated with the Manhattan index, as implemented in the PAST software v.1.93^70^.

### Leaf rust seedling response assays in 185 wheat genotypes

In the second experiment, phenotyping evaluation of an AM panel consisting of 185 worldwide diverse wheat genotypes (Table S2) was carried out at the Cereal Research Department, Seed and Plant Improvement Institute (SPII), Karaj, Iran, in 2018–2019. Wheat genotypes were tested at the seedling stage under greenhouse conditions using a randomized complete block design with two replications against 10 *Pt* races. The *Pt* races were chosen according to different virulence patterns (Table 4) of isolates from distinct geographical regions based on the results of the first experiment. Experimental procedures for inoculation, incubation and disease assessment were the same as those described for race identification in near-isogenic Thatcher lines, using a 0-to-4 scaling system as described previously^24,68^.

**Table 4:** Physiological race and collection site of 10 *Pucnina. triticina* isolates used for phenotypic assessment at the seedling stage on 185 wheat genotypes.

Race	Isolate	Origin (Province/City)
MTTTS	LR15	Khuzestan/Shavoor
PKRQS	LR2	Khuzestan/Dezful
PJTSS	LR59	Ardabil/Ardabil
MFHPS	LR42	Khorasan Razavi/Mashhad
MJTTS	LR35	Mazandaran/Behshahr
FKTPS-1	LR47	Khorasan Razavi/Neishaboor
CTTPR	LR63	Mazandaran/Kalardasht
FKTPS-2	LR16	Khuzestan/Ahvaz
FSRRS	LRG32	Golestan/Gorgan
BRTRS	LR45	Lorestan/Khoram Abad

### Wheat germplasm genotyping using DArTseq platform

A diversity panel of 185 wheat genotypes was grown in a controlled greenhouse. Young leaves from 10-day-old seedlings were used for DNA extraction following the protocol recommended by Diversity Array Technology (DArT) company and whole wheat genotypes were genotyped with the wheat DArTseq platform using the Pst1 complexity reduction method as described before^71^. DArTseq markers were filtered to retain markers with known chromosomal position, markers with ≤ 20% missing data and minor allele frequency (MAF) ≥ 5%^72^.

### Genetic diversity, population structure and linkage disequilibrium (LD)

The genetic diversity and population structure of 185 wheat genotypes were previously described^73^. Briefly, Cluster analysis of diversity panel estimated in DARwin ver. 5.0 software using the Unweight Neighbor-Joining (UNJ) algorithm. Pairwise LD between markers was measured as r^2^ by plotting the r^2^ against the pairwise genetic distance between markers^74,75^. The graphical LD decay was imputed by the GAPIT R package^76^.

Population structure of the 185 wheat genotypes was performed in STRUCTURE 2.1 using the Bayesian clustering algorithm with a burn-in period at 10,000 interactions followed by 10,000 replication of Markov Chain Monte Carlo (MCMC)^77^.

### Genome-wide association analysis for seedling leaf rust resistance

To meet the data format required for GWAS analysis, infection types (IT) data were converted into a linearized scale (LS) of 0–9 scale as described^78^. ITs were converted as follows: 0, 1^−^, 1, 1^+^, 2^−^, 2, 2^+^, 3^−^, 3 and 3^+^ were coded as 0, 1, 2, 3, 4, 5, 6, 7, 8 and 9, respectively. The IT symbol “;” and 4 converted to 0 and 9, respectively. Mesothetic reaction types X −, X, and X + were converted to linearized scores of 4, 5, and 6, respectively. The BLUE value of linearized scale (0–9) for all isolates was calculated using the PROC MIXED procedure in SAS v9.3. In the model, the genotype considered as a fixed effect and replication (block) considered as random effect. These BLUE values were then used for broad sense heritability estimates and correlations between isolates, cluster analysis of wheat genotypes and also to perform GWAS^73^. Genome-wide association mapping (GWAS) analysis was conducted in the R package Genome Association and Prediction Integrated Tool (GAPIT)^76^ using all 21,773 mapped polymorphic DArTseq markers.

Association analysis for each *Pt* race was conducted using the FarmCPU model^35,79^. Association results of the FarmCPU model were compared with association models like as GLM, MLM, CMLM and Super-MLM models and finally, this model provided a robust model for association mapping of resistance genes against Pt races, which effectively controls both false positives and false negatives^80^.

The quantile–quantile (Q-Q) plot of each *Pt* race was drawn using the observed and expected log_*10*_ *P* values. Marker–trait associations (MTAs) were selected if the significant markers cross the false discovery rate threshold (P = 0.05) and a uniform threshold level of *P-value* ≥ *0.0001 (− log10 P* = *4.00)*. Significant MTAs associated with *Pt* races were ordered according to their genetic map positions in a high-resolution DArT-seq consensus map (version 4.0), provided by Dr. Andrzej Kilian (Diversity Arrays Technology Pty Ltd, Canberra, Australia). The identified QTLs and catalogued *Lr* genes^81^ were projected onto the wheat integrated consensus map^38^ and their positions were compared with previously known *Lr* genes and 393 QTLs from 50 QTL mapping studies^39^. Each QTL was considered new if its position was ≥ 10 cM from previously reported *Lr* genes or QTLs^12^. In order to find the candidate genes linked to MTAs, the physical position of these markers was taken to Ensembl using IWGSC RefSeq v1.0 genome and ~ 2.5 Mb flanking each marker was considered for annotated genes^19^.

### Ethics approval and consent to participate

All the plant materials provided by Iranian Seed and Plant Improvement Institute (SPII) and were in compliance with relevant institutional, national, and international guidelines and legislation.

## Supplementary Information

Supplementary Figure S1.

Supplementary Figure S2.

Supplementary Figure S3.

Supplementary Figure S4.

Supplementary Figure S5.

Supplementary Table S1.

Supplementary Table S2.

## Data Availability

The plant materials used during the current study are available from the corresponding author on reasonable request. The DArTseq datasets generated and analyzed during the current study are available in the Figshare repository: https://figshare.com/articles/dataset/DArTseq-Data_xlsx/21967460.
