# Supplementary material for: Physiological specialization of Puccinia triticina and genome-wide association mapping provide insights into the genetics of wheat leaf rust resistance in Iran
Source: Sci Rep. 2023 Mar 16;13:4398. doi: 10.1038/s41598-023-31559-y (PMC10020449; doi:10.1038/s41598-023-31559-y)
Supplement: Supplementary file 1 — Supplementary Figure S1. [file 41598_2023_31559_MOESM1_ESM.docx]

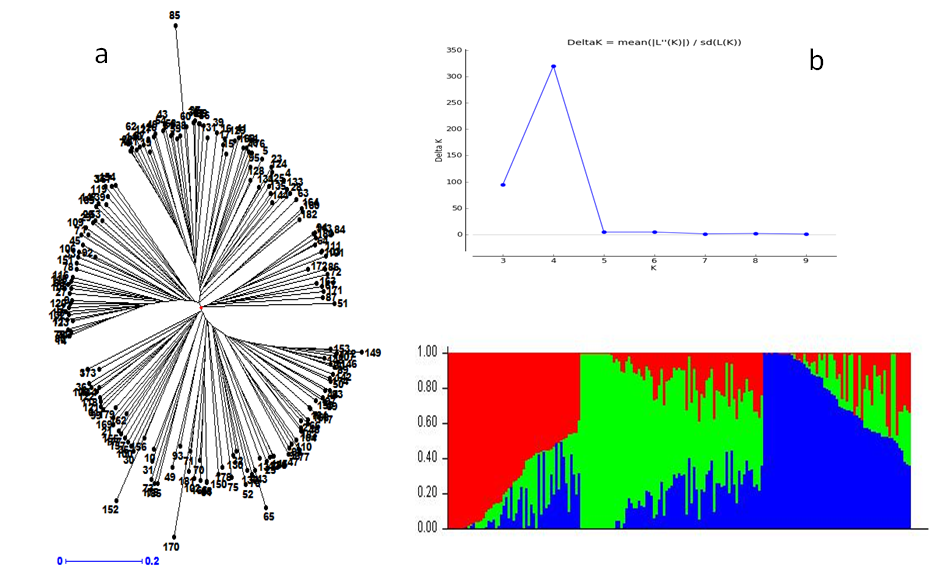


**Figure S1**. Cluster analysis and population-based structure analysis of 185 wheat genotypes using DArTseq markers
