# Supplementary material for: Physiological specialization of Puccinia triticina and genome-wide association mapping provide insights into the genetics of wheat leaf rust resistance in Iran
Source: Sci Rep. 2023 Mar 16;13:4398. doi: 10.1038/s41598-023-31559-y (PMC10020449; doi:10.1038/s41598-023-31559-y)
Supplement: Supplementary file 2 — Supplementary Figure S2. [file 41598_2023_31559_MOESM2_ESM.docx]

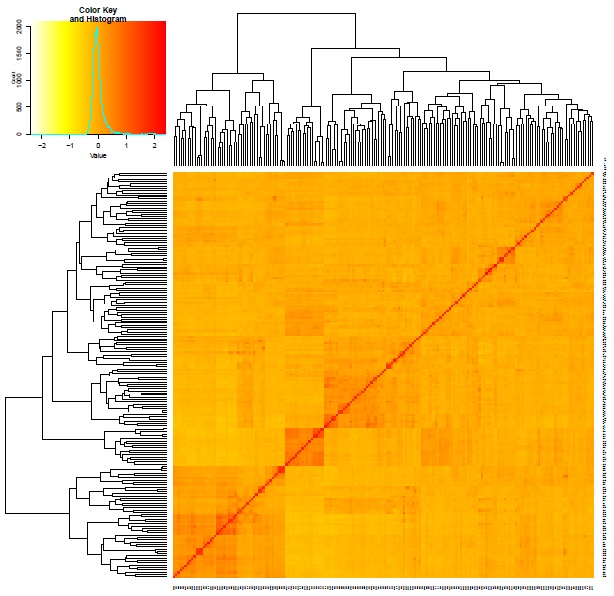


**Figure S2**. Heatmap plot of kinship matrix displaying relationships of 185 wheat genotypes based on DArTseq markers.
