# Supplementary material for: Physiological specialization of Puccinia triticina and genome-wide association mapping provide insights into the genetics of wheat leaf rust resistance in Iran
Source: Sci Rep. 2023 Mar 16;13:4398. doi: 10.1038/s41598-023-31559-y (PMC10020449; doi:10.1038/s41598-023-31559-y)
Supplement: Supplementary file 3 — Supplementary Figure S3. [file 41598_2023_31559_MOESM3_ESM.docx]

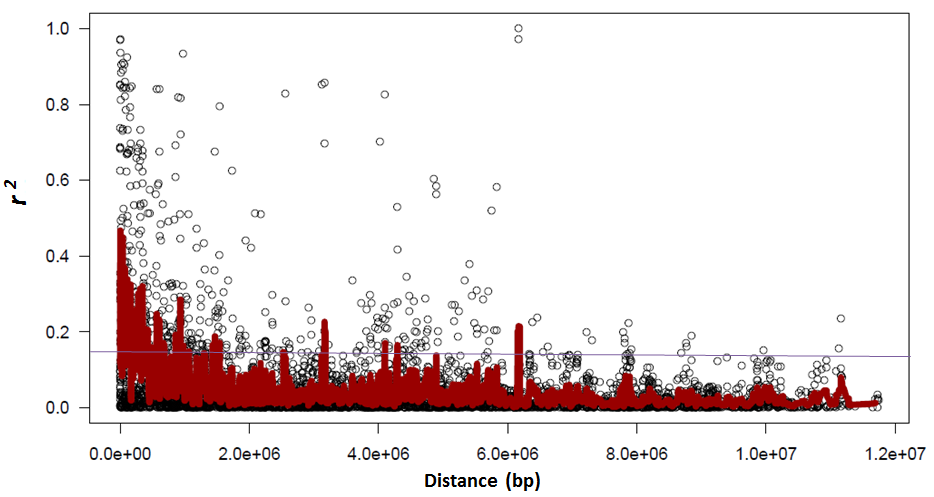


**Figure S3**. Linkage disequilibrium (LD) measured r^2^ plotted vs. the physical map (bp) between pairs of DArTseq markers in a panel of 185 wheat genotypes
