# Supplementary material for: Physiological specialization of Puccinia triticina and genome-wide association mapping provide insights into the genetics of wheat leaf rust resistance in Iran
Source: Sci Rep. 2023 Mar 16;13:4398. doi: 10.1038/s41598-023-31559-y (PMC10020449; doi:10.1038/s41598-023-31559-y)
Supplement: Supplementary file 4 — Supplementary Figure S4. [file 41598_2023_31559_MOESM4_ESM.pdf]

Figure S4. Manhattan plots showing significant DArTseq markers associated with resistance to different *Puccinia triticina* races. The horizontal line indicates the arbitrary threshold of significance of *–log10(P)* ≥ *4.0* corresponding to a *P-value* ≤ 0.0001 that was accepted as significant markers-trait association.

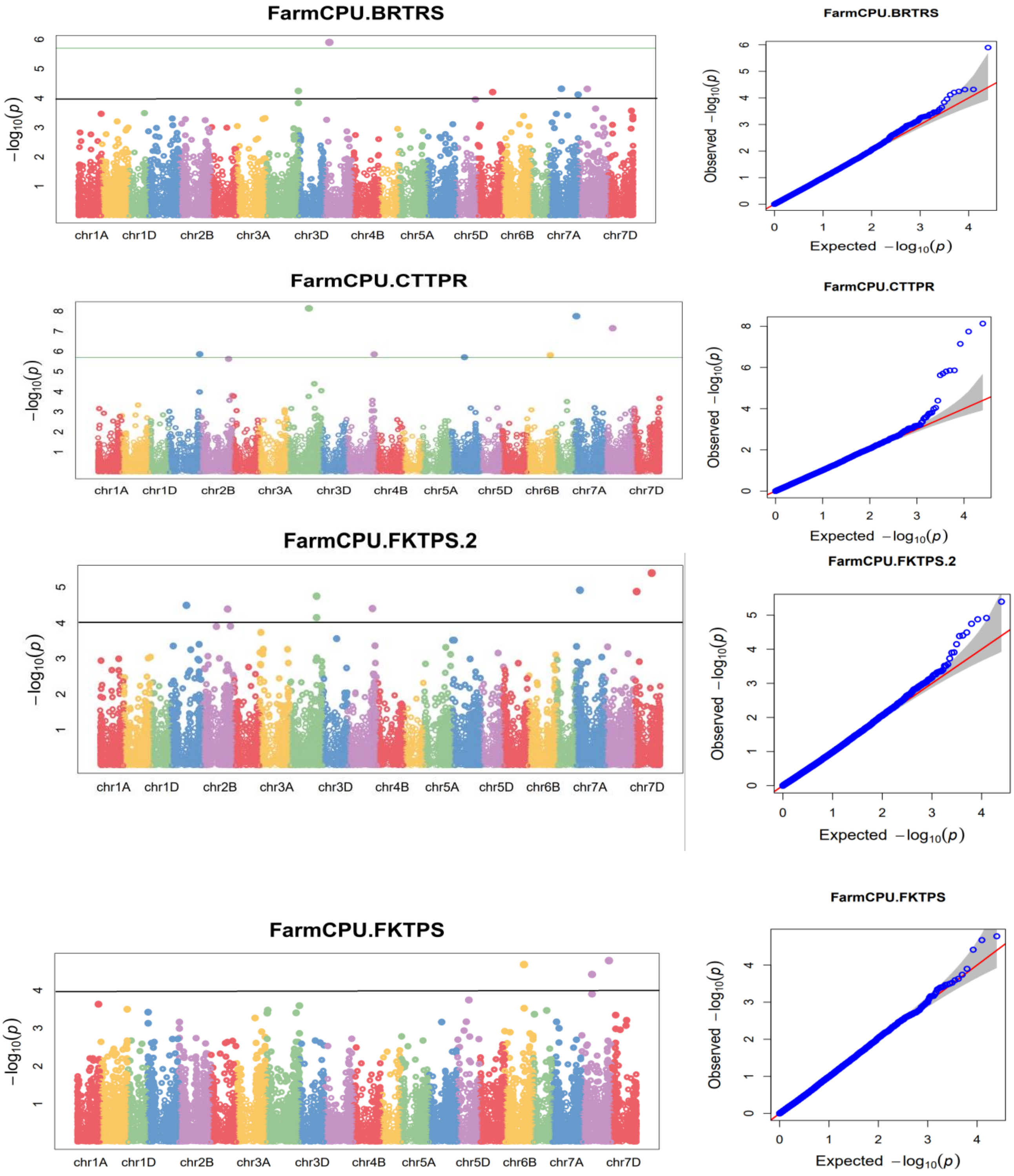

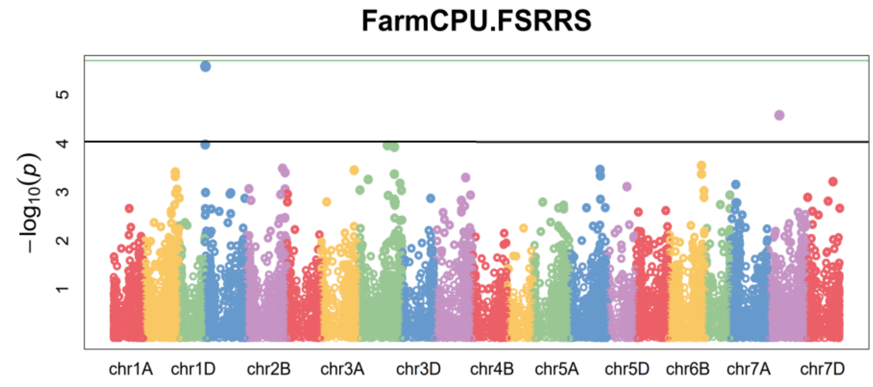
FarmCPU.FSRRS
−log10(p)
chr1A chr1D chr2B chr3A chr3D chr4B chr5A chr5D chr6B chr7A chr7D
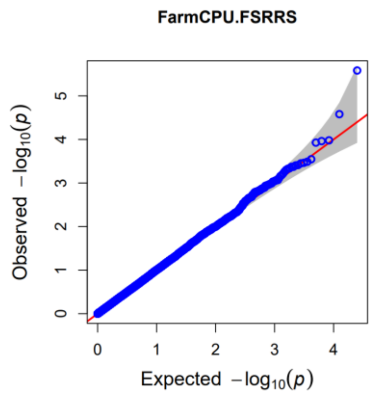
FarmCPU.FSRRS
Observed −log10(p)
Expected −log10(p)
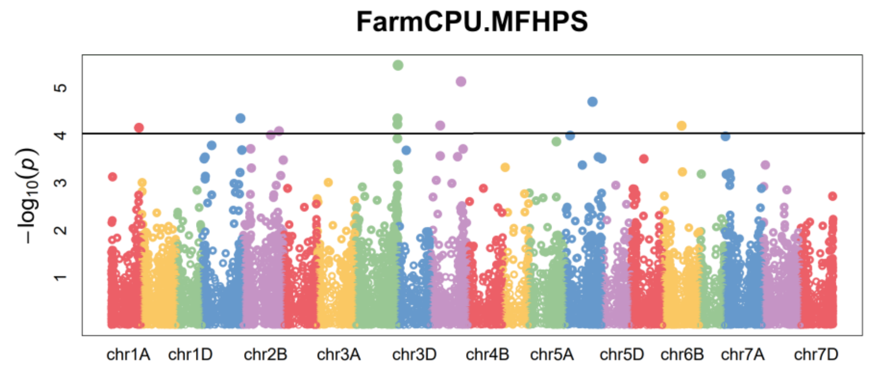
FarmCPU.MFHPS
−log10(p)
chr1A chr1D chr2B chr3A chr3D chr4B chr5A chr5D chr6B chr7A chr7D
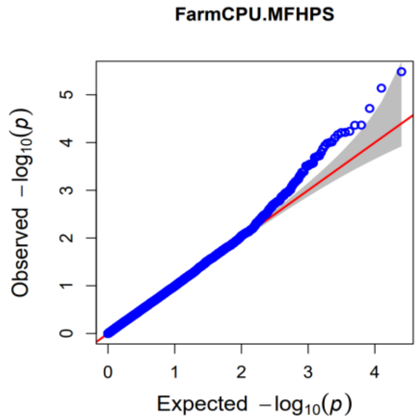
FarmCPU.MFHPS
Observed −log10(p)
Expected −log10(p)
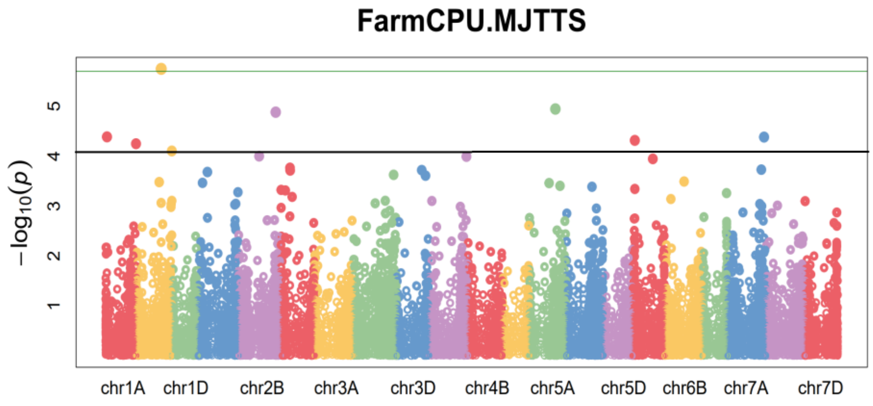
FarmCPU.MJTTS
−log10(p)
chr1A chr1D chr2B chr3A chr3D chr4B chr5A chr5D chr6B chr7A chr7D
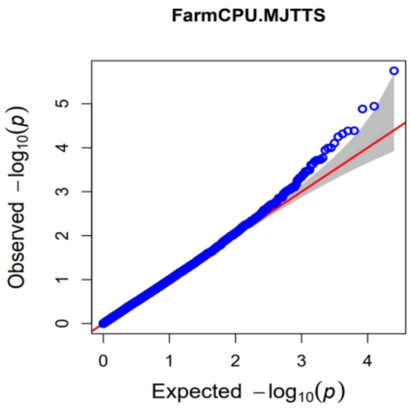
FarmCPU.MJTTS
Observed −log10(p)
Expected −log10(p)

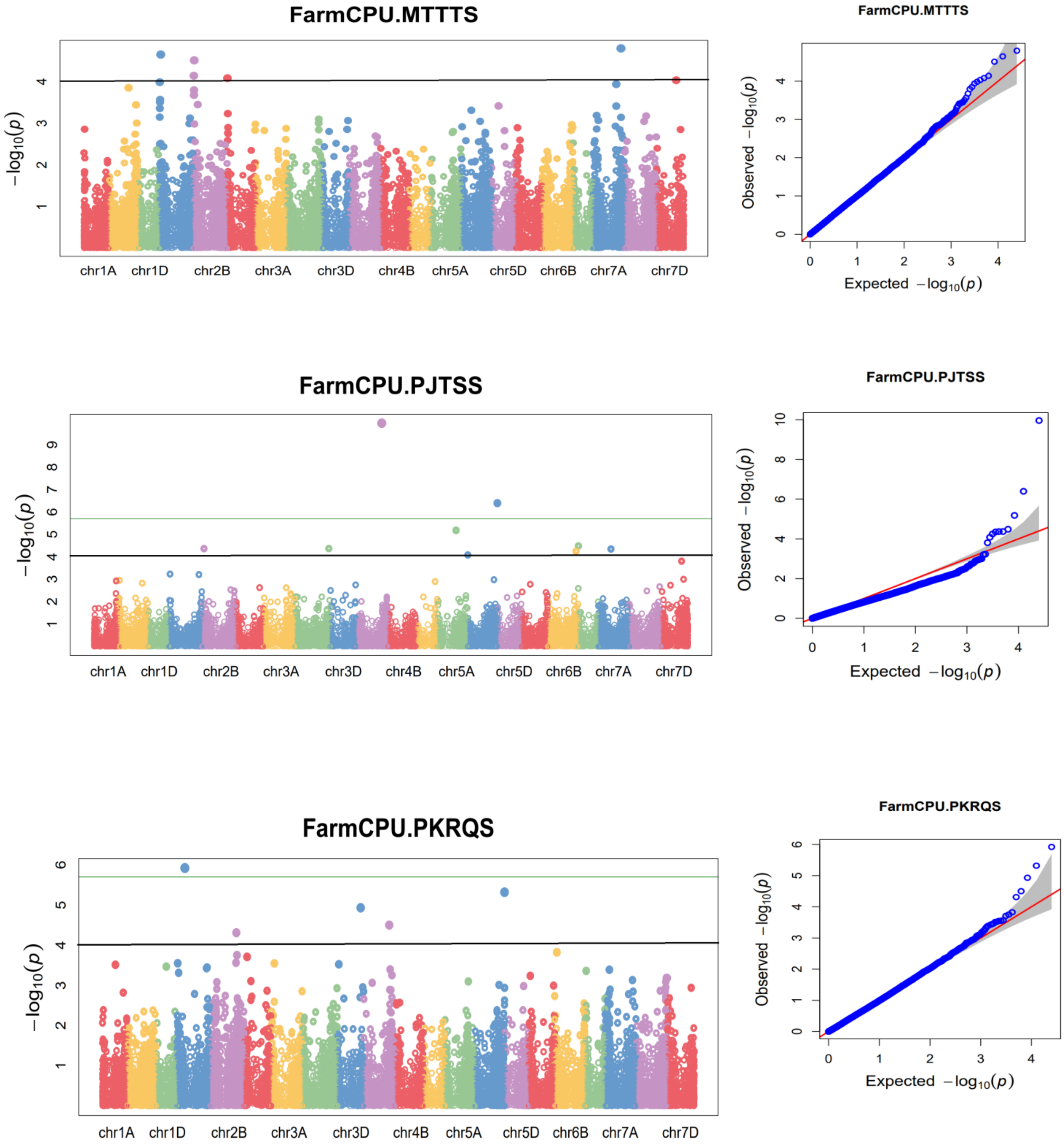
FarmCPU.MTTTS
−log10(p)
chr1A chr1D chr2B chr3A chr3D chr4B chr5A chr5D chr6B chr7A chr7D
FarmCPU.MTTTS
Observed −log10(p)
Expected −log10(p)
FarmCPU.PJTSS
−log10(p)
chr1A chr1D chr2B chr3A chr3D chr4B chr5A chr5D chr6B chr7A chr7D
FarmCPU.PJTSS
Observed −log10(p)
Expected −log10(p)
FarmCPU.PKRQS
−log10(p)
chr1A chr1D chr2B chr3A chr3D chr4B chr5A chr5D chr6B chr7A chr7D
FarmCPU.PKRQS
Observed −log10(p)
Expected −log10(p)
