# Supplementary material for: Physiological specialization of Puccinia triticina and genome-wide association mapping provide insights into the genetics of wheat leaf rust resistance in Iran
Source: Sci Rep. 2023 Mar 16;13:4398. doi: 10.1038/s41598-023-31559-y (PMC10020449; doi:10.1038/s41598-023-31559-y)

**Figure S5**. Genomic regions of leaf rust resistance QTLs in 185 wheat genotypes panel projected on the common integrated smap developed by Macafferri et al. (2015). Location of major leaf rust resistance genes/QTLs from 50 QTL mapping studies retrieved by Aduragbemi and Soriano (2021).


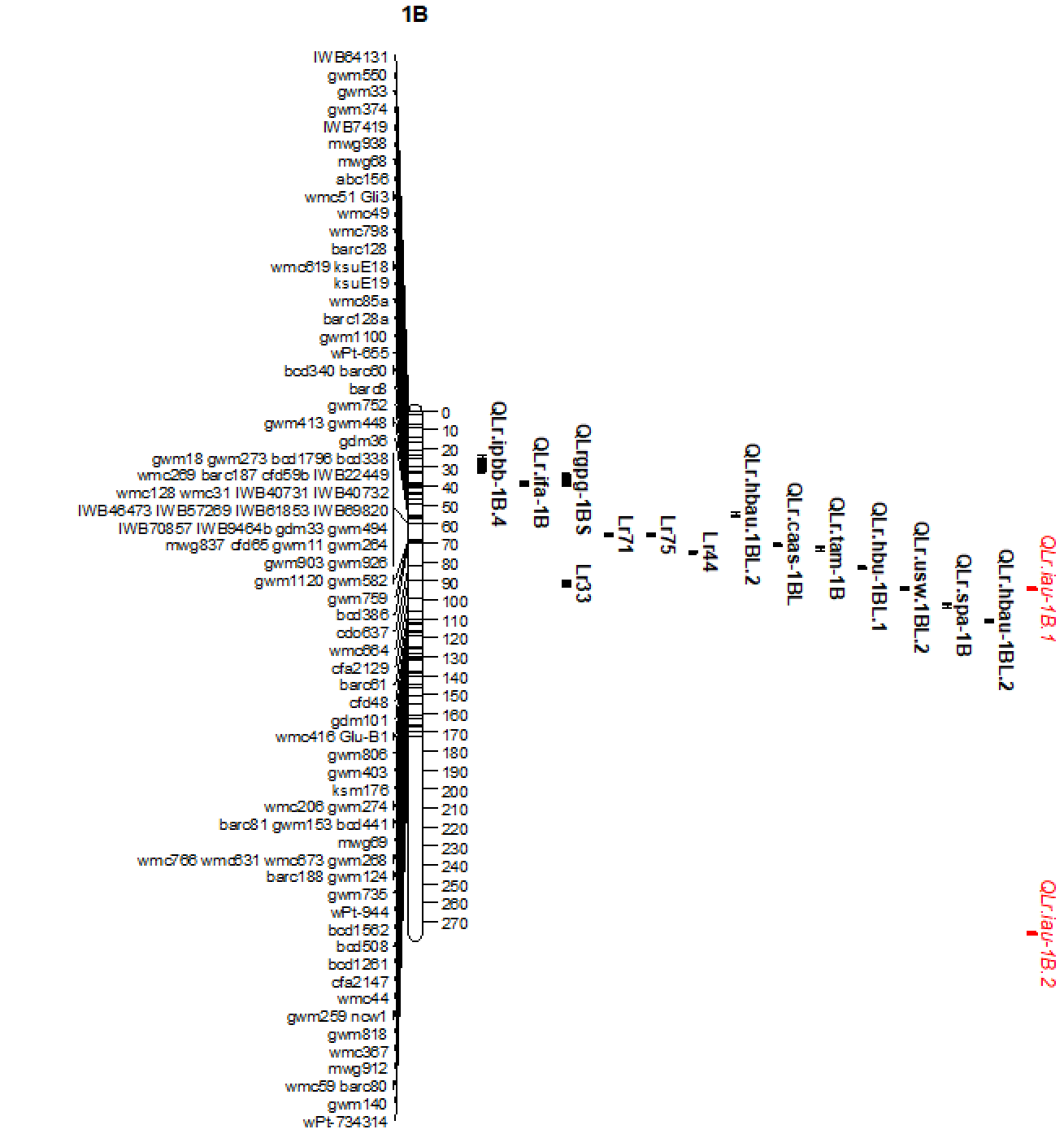


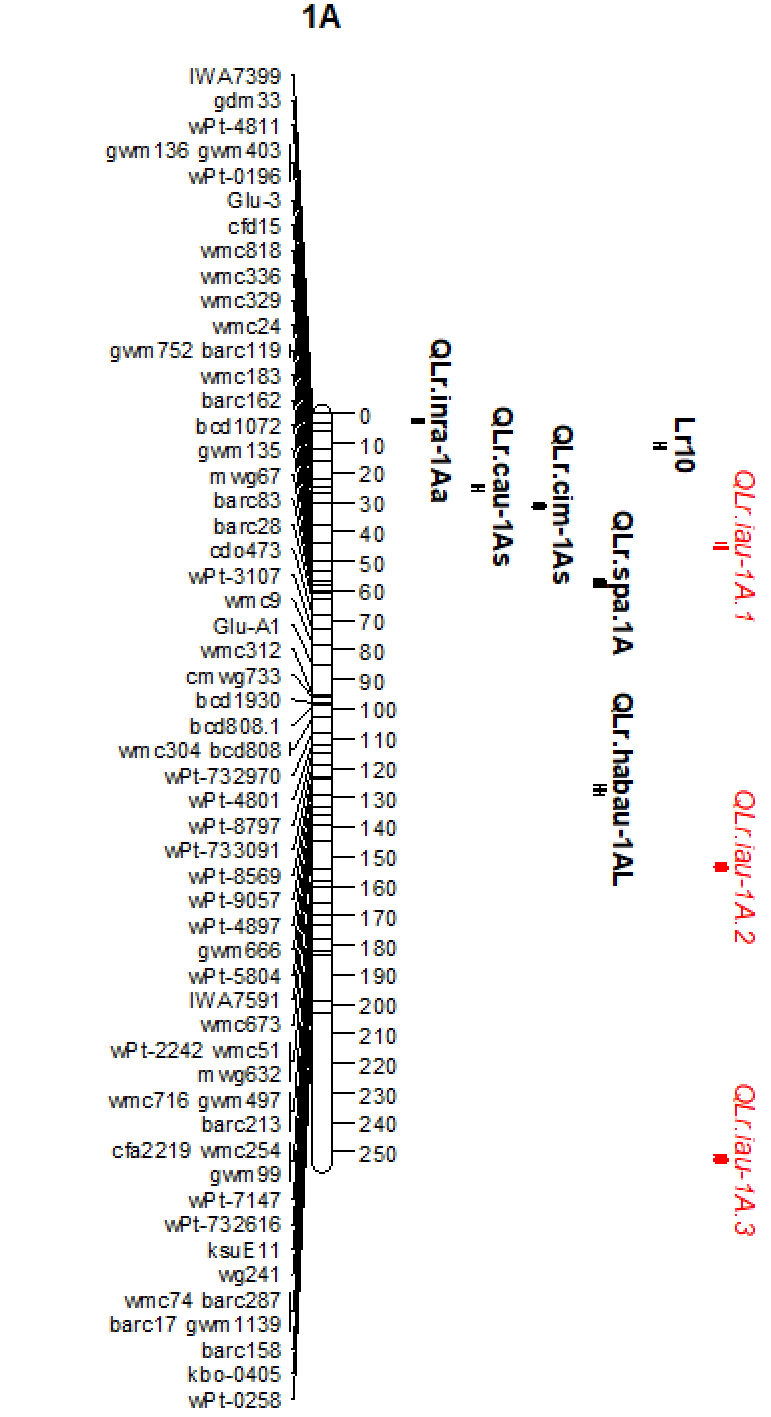


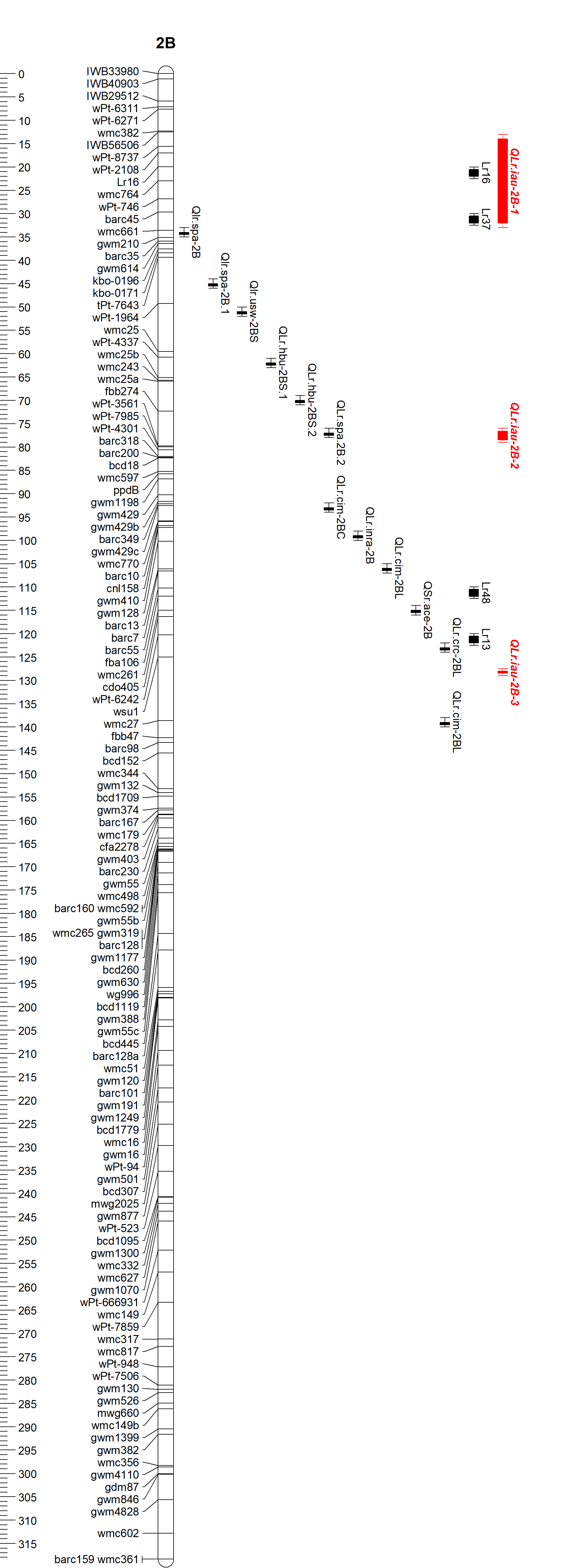


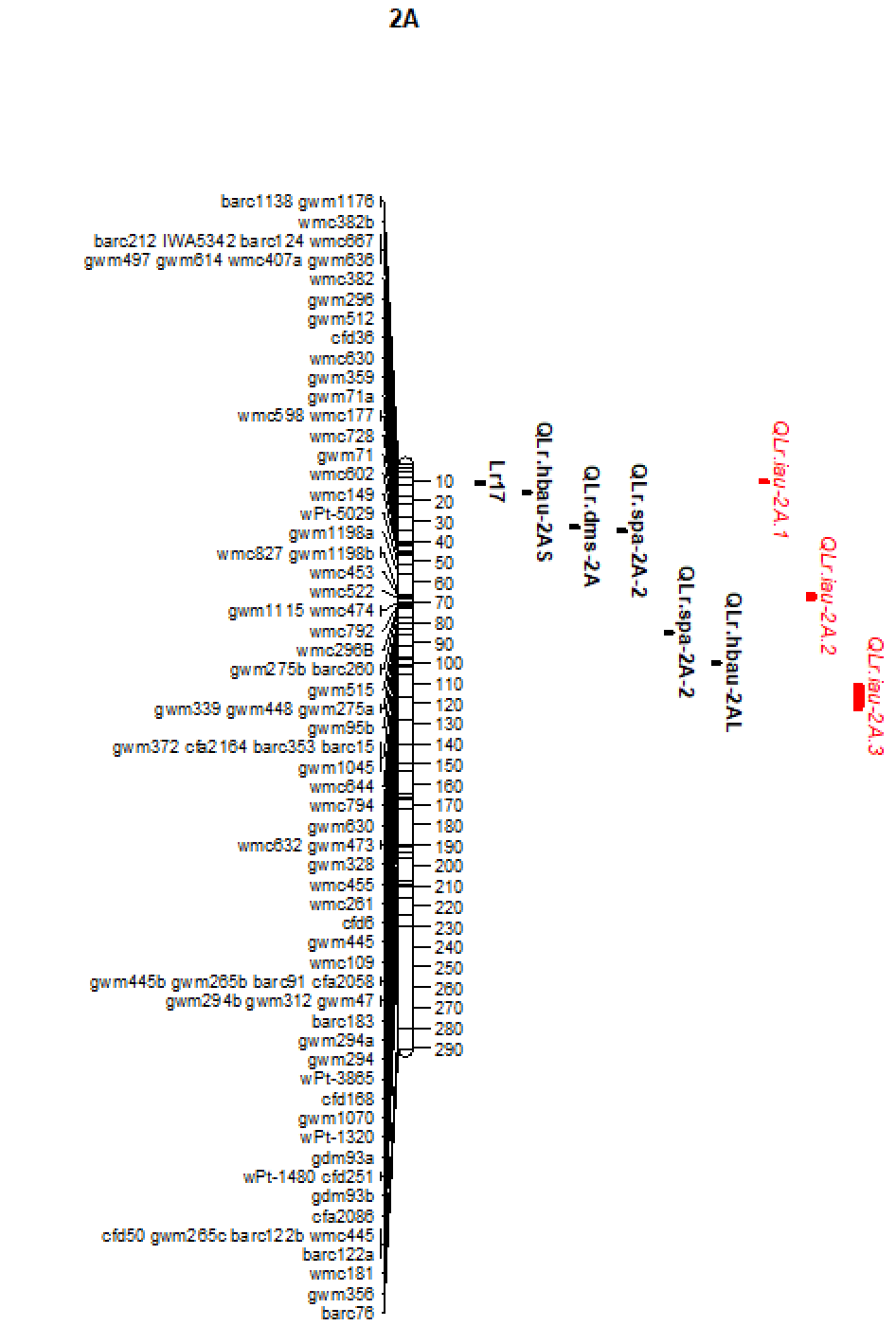


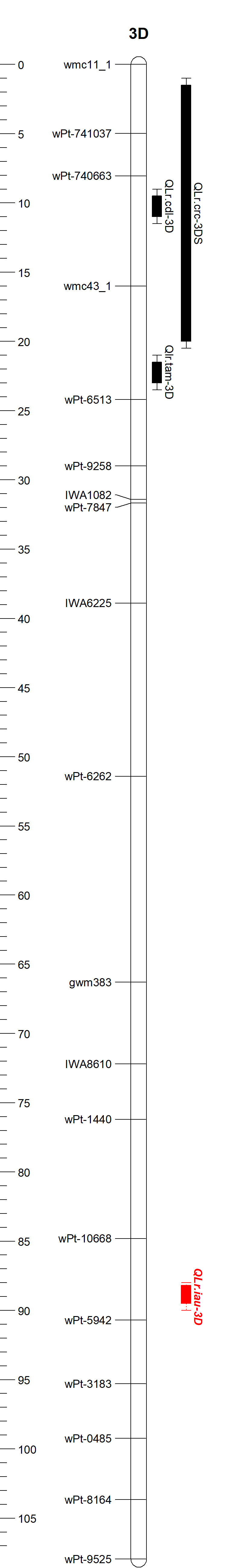


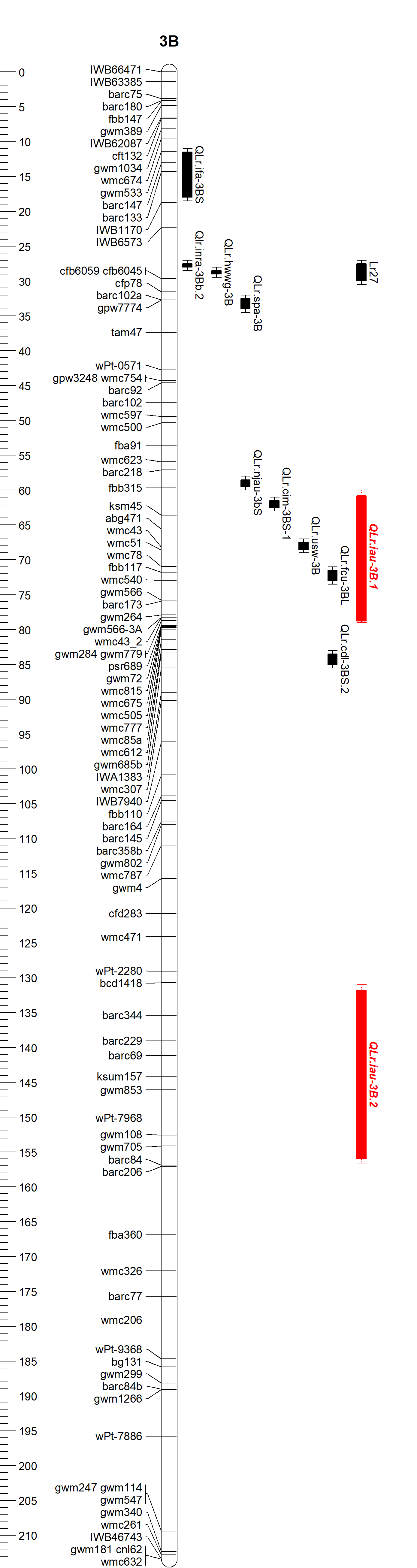


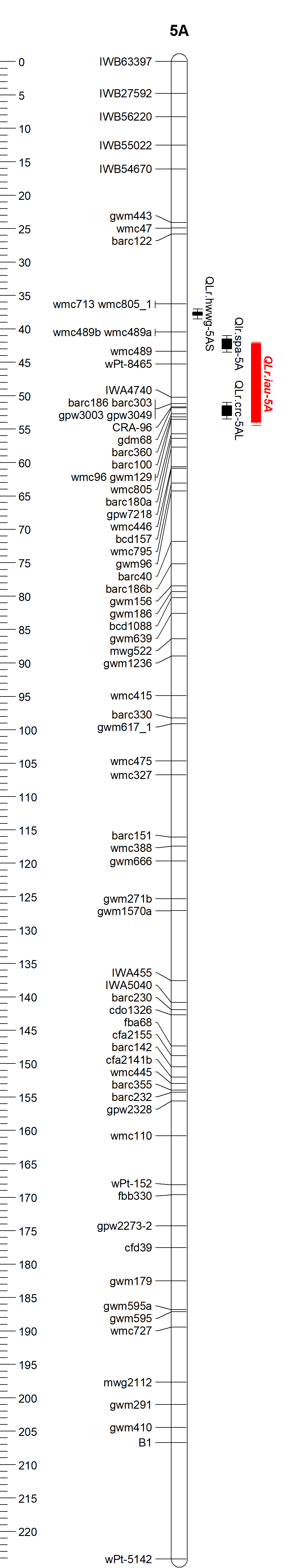


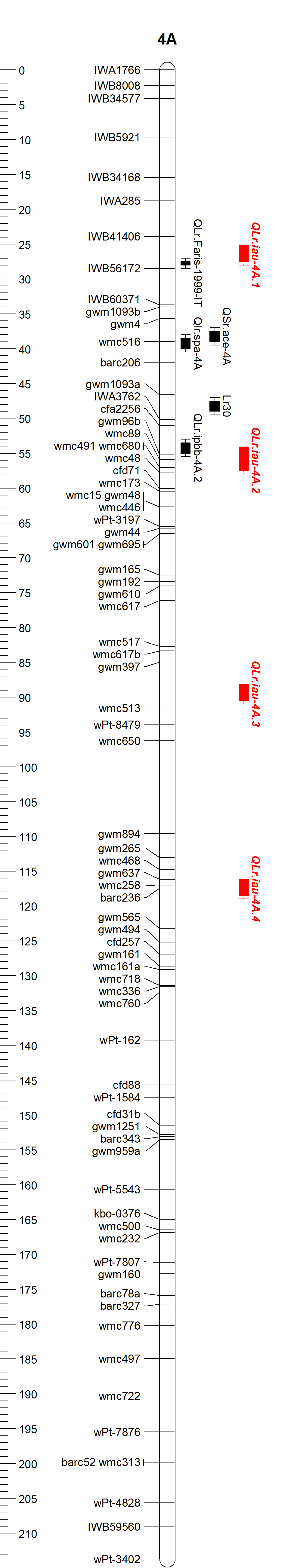


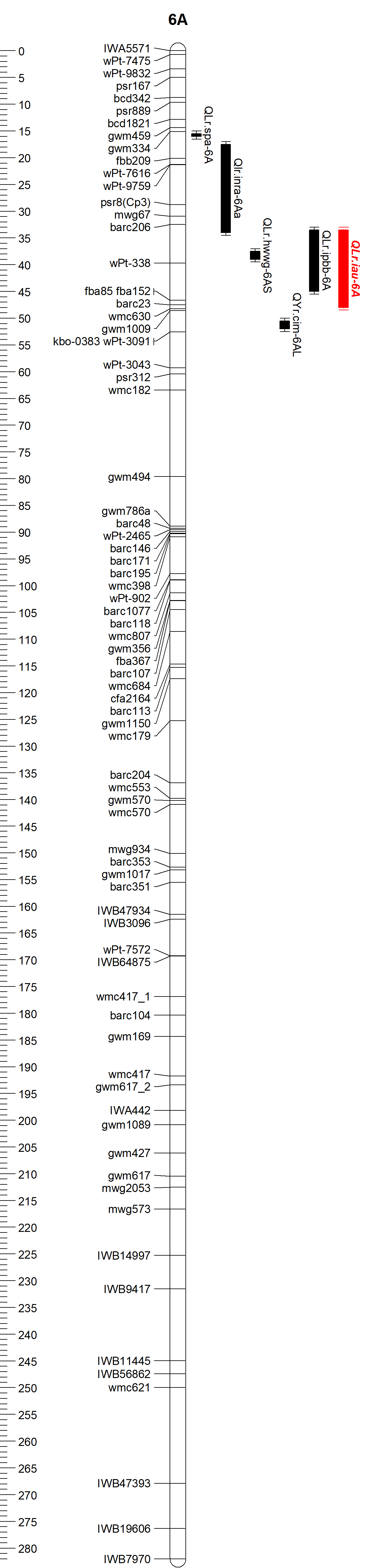


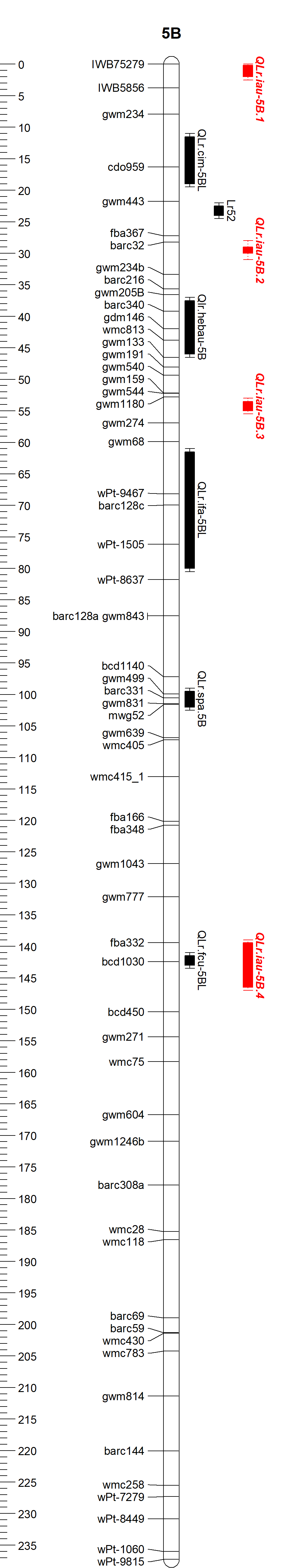


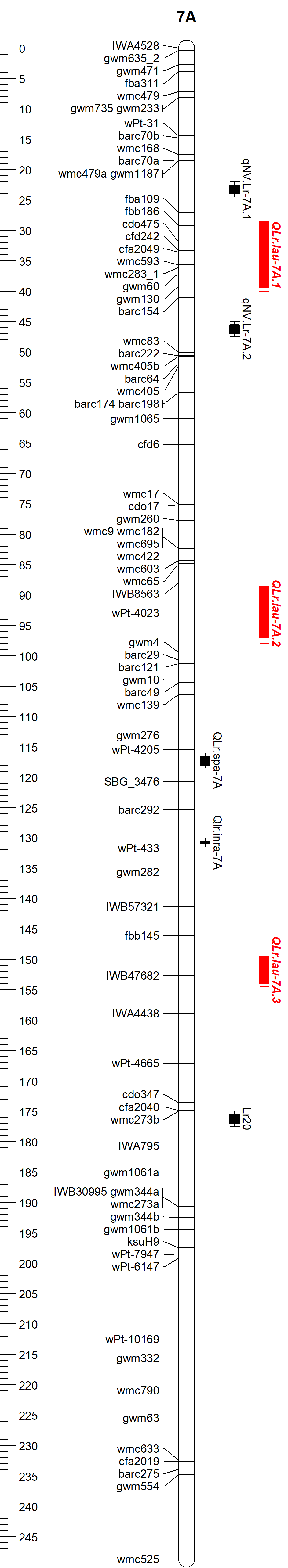


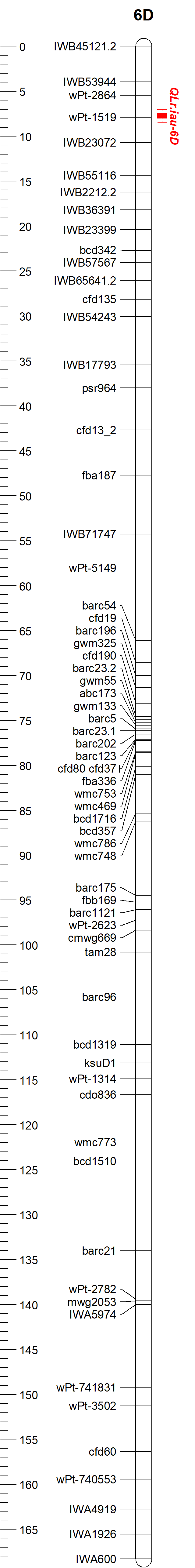


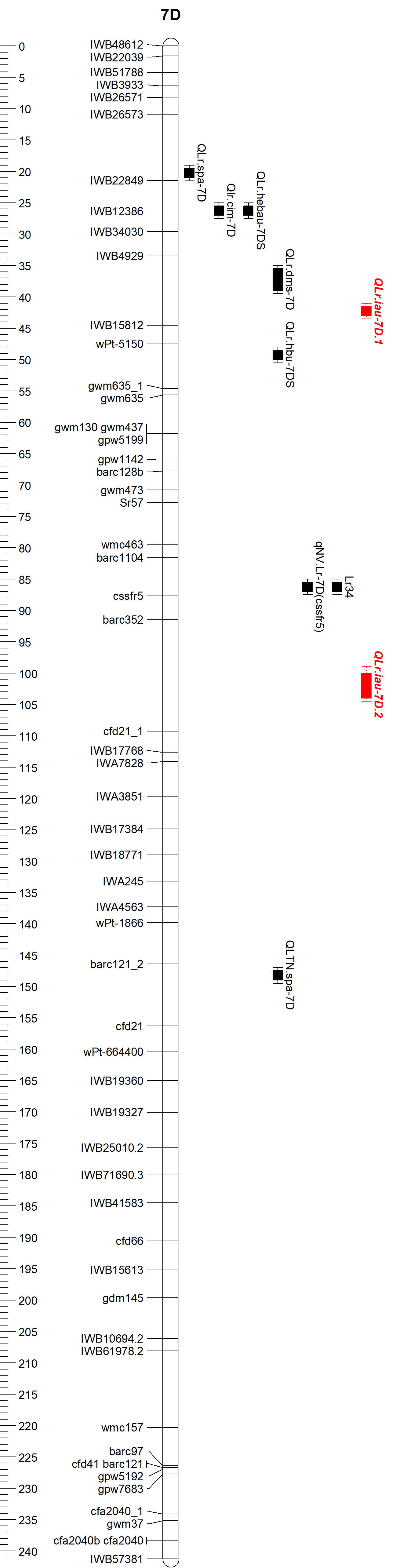


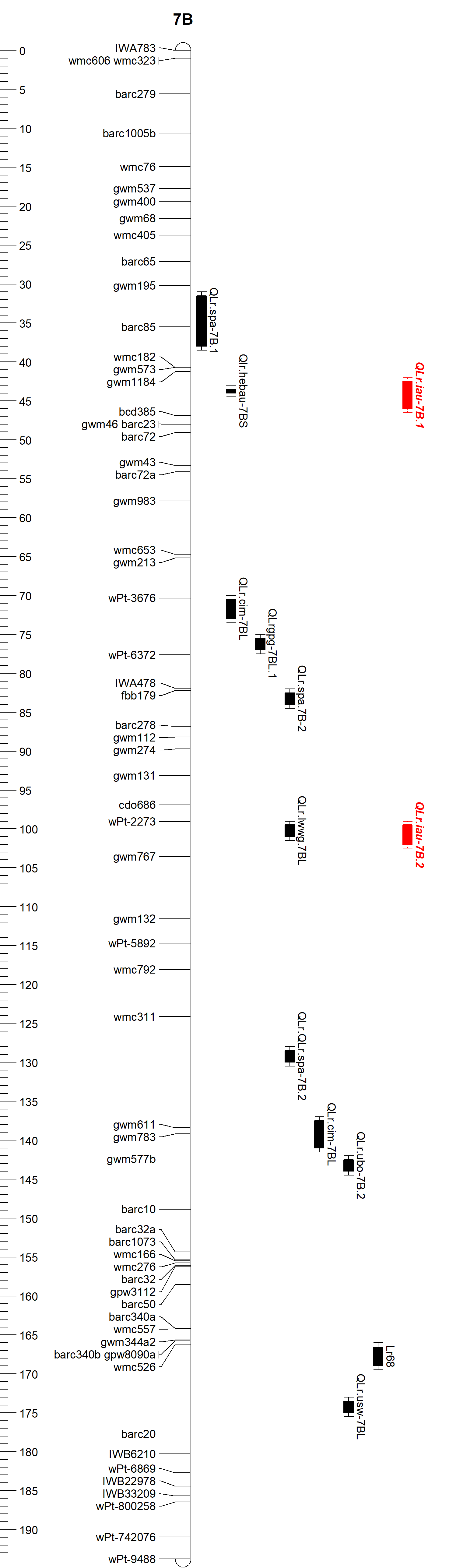

Supplement: Supplementary file 5 — Supplementary Figure S5. [file 41598_2023_31559_MOESM5_ESM.docx]
